# Proteomic analysis of plasma exosomes to differentiate malignant from benign pulmonary nodules

**DOI:** 10.1186/s12014-019-9225-5

**Published:** 2019-02-02

**Authors:** Muyu Kuang, Xiaoting Tao, Yizhou Peng, Wenjing Zhang, Yafang Pan, Lei Cheng, Chongze Yuan, Yue Zhao, Hengyu Mao, Lingdun Zhuge, Zhenhua Zhou, Haiquan Chen, Yihua Sun

**Affiliations:** 1 Department of Thoracic Surgery, Fudan University Shanghai Cancer Center, Shanghai, China; 2 Department of Oncology, Shanghai Medical College, Fudan University, Shanghai, China; 3 Huadong Hospital, Fudan University, Shanghai, China; 4 Shanghai Institute of Biochemistry and Cell Biology, Shanghai, China; 5 Cancer Institute, Collaborative Innovation Center for Cancer Medicine, Fudan University Shanghai Cancer Center, Shanghai, China; 6 Department of Orthopaedic Oncology, Changzheng Hospital, Naval Military Medical University (The Second Military Medical University), Shanghai, China; 7 Present Address: Department of Thoracic Surgery, Fudan University Shanghai Cancer Center, No. 270, Dongan Road, Shanghai, 200030 China

## Abstract

**Background:**

It is difficult to distinguish benign pulmonary nodules (PNs) from malignant PNs by conventional examination. Therefore, novel biomarkers that can identify the nature of PNs are needed. Exosomes have recently been identified as an attractive alternative approach since tumor-specific molecules can be found in exosomes isolated from biological fluids.

**Methods:**

Plasma exosomes were extracted via the exoEasy reagent method. The major proteins from plasma exosomes in patients with PNs were identified via labelfree analysis and screened for differentially expressed proteins. A GO classification analysis and KEGG pathway analysis were performed on plasma exosomal protein from patients with benign and malignant PNs.

**Results:**

Western blot confirmed that protein expression of CD63 and CD9 could be detected in the exosome extract. Via a search of the human Uniprot database, 736 plasma exosome proteins from patients with PNs were detected using high-confidence peptides. There were 33 differentially expressed proteins in the benign and malignant PNs. Of these, 12 proteins were only expressed in the benign PNs group, while 9 proteins were only expressed in the malignant PNs group. We further obtained important information on signaling pathways and nodal proteins related to differential benign and malignant PNs via bioinformatic analysis methods such as GO, KEGG, and String.

**Conclusions:**

This study provides a new perspective on the identification of novel detection strategies for benign and malignant PNs. We hope our findings can provide clues for the identification of benign and malignant PNs.

**Electronic supplementary material:**

The online version of this article (10.1186/s12014-019-9225-5) contains supplementary material, which is available to authorized users.

## Introduction

Hundreds of thousands of patients are diagnosed with pulmonary nodules (PNs) each year, and this number is on the rise [1, 2]. In China, due to the improvement of medical standards, more people routinely undergo physical examinations and lung computed tomography (CT) examinations, and many of these patients are diagnosed with PNs. Identifying the nature of these PNs is of great significance for the development of the patient’s treatment plan. Although low-dose computed tomography (LDCT) screening was widely employed clinically, a high prevalence of false positives was found in the early diagnosis of lung cancer [3]; due to this, there was no consensus on how to manage these PNs. On the other hand, the high prevalence of false positives for PNs may lead to over-treatment, anxiety induction and excessive use of invasive procedures. There is a critical need to develop less invasive and less expensive techniques with high sensitivity and specificity to aid in monitoring patients with PNs for either benign conditions or early-stage cancer.

Exosomes are 30–150 nm diameter vesicles released during the fusion of multivesicular endosomes with the plasma membrane [4]. Different size of exosomes had unique glycosylation, protein, lipid, and DNA and RNA profiles and biophysical properties [5], and extracellular vesicle heterogeneity can be defined by variation in cargo between and within each size class, as well as by variation in size [6]. These vesicles have been implicated in a number of different tumor physiological processes as rich reservoirs of tumor-specific proteins and biomarkers for cancer detection and progression. A better understanding of the contents of exosomes is crucial to the assessment of the probability of malignancy of PNs. Exosomes secreted by PNs can be isolated from the blood for further proteomic analysis. With this in mind, we conducted a comparative analysis of proteins in circulating exosomes collected from patients with PNs. To our knowledge, our study is the first to use high-throughput proteomic analysis to compare benign and malignant PNs-derived exosomes in an Asian population. We hope that our findings will bring new ideas and perspectives for the differentiation of benign and malignant PNs and provide useful tools for the early detection and diagnosis of lung cancer.

## Materials and methods

### Patients and ethics statement

All samples were obtained from the Department of Thoracic Surgery, Fudan University Shanghai Cancer Center, after written informed consent was obtained. The study was performed in agreement with the Helsinki Declaration and approved by the Ethical Committee at the Fudan University Shanghai Cancer Center. For plasma analysis, we included 40 patients who were newly diagnosed with PNs by CT. Fresh whole blood samples were collected before each operation in tubes containing EDTA, followed by exosome isolation. The final diagnoses were made according to pathological examinations performed after each operation. 10 of the 40 patients were diagnosed with benign diseases and the rest with non-small cell lung cancer (NSCLC) according to the pathological diagnosis.

### Exosome isolation and mass spectrometry

Plasma samples were pass through a 0.8 μm filter to remove additional cellular fragments and cell debris before exosome isolation (Millipore Millex). Exosomes were collected using the exoEasy Maxi Kit (Qiagen) [7]. A total of 10 ml buffer XWP were added and centrifuge at 5000*g* for 5 min to wash exosomes. We used 400 μL of Buffer XE (Qiagen) to dissolve the exosomes, centrifuged at 5000*g* for 5 min and then collected the eluate. All steps followed the manufacturer’s instructions. Purified exosomes were then stored at − 80 °C until use.

### Western blotting

Protein concentration was quantified by BCA kit (Bio-red), and protein concentration data were shown as Additional file 1: Table S1. Protein was resolved by SDS-PAGE and transferred to PVDF membranes using a semi-dry transfer as previously described [8]. Membranes were blocked in 5% non-fat powdered milk in TBST and probed with antibodies. Proteins were detected using X-ray film and enhanced chemiluminescence reagents (Thermo Pierce ECL Western Blotting Substrate).

### Transmission electron microscope

Twenty microliters of exosomes was layered and absorbed on a formvar-coated 200 mesh copper grid and stained with 2% uranyl acetate. The sample was imaged using an FEI Tecnai G2 Spirit transmission electron microscope equipped with a thermionic Lab6 filament and operated at an acceleration of 80 kV.

### Sample preparation for a label-free experiment

For exosome lysis, 100 μL of lysis buffer (4% SDS, 100 mM DTT, 150 mM Tris–HCl pH 8.0) was added. The samples were ultrasonicated, boiled for 10 min then centrifuged at 13,400 rpm for 30 min. The supernatant was collected and quantified using a BCA Protein Assay Kit (Bio-Rad, USA). Samples were divided into six groups before label-free mass spectrometry analyses (Fig. 2a): B1 (3 samples), B2 (4 samples), B3 (3 samples) for benign group and M1 (10 samples), M2 (10 samples), M3 (10 samples) for malignant group. Isopyknic samples were polled.

### SDS-PAGE of proteins

Twenty micrograms of protein from each group was used for SDS-PAGE analysis. 5× loading buffer was used, followed by 12.5% SDS-PAGE for 60 min at 15 mA. Coomassie blue staining was applied for detecting proteins (Additional file 1: Figure S1).

### Protein FASP Digestion

Protein digestion was performed according to the FASP procedure described by Wisniewski et al. [9]. As such, 100 mM DTT was added to the sample followed by boiling for 5 min and then cooling to room temperature. The DTT and other low-molecular-weight components were removed using 200 μl of UA buffer (8 M Urea, 150 mM Tris–HCl pH 8.0) by repeated ultrafiltration facilitated by centrifugation at 14,000 g for 15 min. Next, 100 μL of 50 mM IAA in UA buffer was added to block reduced cysteine residues, and the samples were incubated for 30 min in darkness. The filter was washed twice with 100 μl of UA buffer and then 100 μl of NH_4_HCO_3_. Finally, the protein suspension was digested with 40 μl of trypsin buffer (Promega) overnight at 37 °C, and the resulting peptides were collected. The peptide material was desalted, and its concentration was estimated by UV light spectral density at 280 nm using an extinction coefficient of 1.1 for the 0.1% (g/l) solution.

### Liquid chromatography (LC)-electrospray ionization (ESI) tandem mass spectrometry (MS/MS) analysis by Q exactive

MS experiments were performed on a Q Exactive mass spectrometer that was coupled to an Easy nLC1000 (Thermo Fisher Scientific). The peptide of each sample was desalted on C18 Cartridges (Empore™ SPE Cartridges C18 (standard density), bed I.D. 7 mm, volume 3 ml, Sigma), then concentrated by vacuum centrifugation and reconstituted in 40 µl of 0.1% (v/v) trifluoroacetic acid. Two micrograms of peptide was loaded onto a C18-reverse phase column in buffer A (2% acetonitrile, 0.1% formic acid) and separated with a linear gradient of buffer B (0.1% formic acid, 84% acetonitrile) at a flow rate of 400 nL/min controlled by IntelliFlow technology for 2 h. MS data were acquired using a data-dependent top-10 method dynamically choosing the most abundant precursor ions from the survey scan (300–1800 m/z) for HCD fragmentation. Survey scans were acquired at a resolution of 70,000 at m/z 200 and resolution for HCD spectra was set to 17,500 at m/z 200. The instrument was run with peptide recognition mode enabled. MS experiments were performed for each sample in triplicate.

### Sequence database searching and data analysis

The MS data were analyzed using MaxQuant software version 1.3.0.5. MS data were used to query the Uniprot_HomoSapiens_154578_20160822.fasta (154578 total entries, downloaded 22/08/16). Label-free quantification was carried out in MaxQuant as previously described [10, 11]. Data from MaxQuant were analyzed by Perseus (1.3.0.4).

### Statistical analyses

Statistical analyses were performed using Bioconductor packages and in-house scripts in the statistical software package R [12]. The technical replicates of the batch-corrected data were averaged, and the resulting data were used for hierarchical clustering analyses. For each identified protein, a linear model was fit to the normalized data containing the two conditions (benign and malignant) as the explanatory variable; for the tissue study, a batch factor was also included in the linear model. Differentially expressed proteins between the benign and malignant groups were detected using a moderated *t* test. Limma (version 3.28.21) 24 and the Benjamini and Hochberg adjustment were applied to control the false discovery rate (FDR) [13]. Proteins were considered to be significantly differentially expressed between the two conditions with multiplying multiples > 1.5 times [14] and an adjusted p value of less than 0.05. An FDR threshold of 0.05 was applied to specify differential abundance for the exosome dataset.

## Results

### Research cohort

In the present study, 40 patients with pulmonary nodules were contained, 30 of whom were diagnosed with malignant and 10 with benign pulmonary nodules. Clinical features were shown in Table 1, there was no significant difference of sex, age, and smoking status between the malignant and benign pulmonary nodule patients.

**Table 1:** Clinicopathologic features of patients

Features	Benign PNs (n = 10)	Malignant PNs (n = 30)	χ^2^	p
Sex			0.034	0.845
Male	4 (40.0%)	13 (43.3%)		
Female	6 (60.0%)	17 (56.7%)		
Age			1.212	0.271
> Median	6 (60.0%)	12 (40.0%)		
≤ Median	4 (40.0%)	18 (60.0%)		
Smoking			0.036	0.850
No	6 (60%)	19 (63.3%)		
Yes	4 (40%)	11 (36.7%)		
Histologic diagnosis				
Lung adenocarcinoma		30 (100%)		
Granuloma	3 (30%)			
Inflammation	3 (30%)			
Hamartoma	1 (10%)			
Dysplasia	2 (20%)			
Other	1 (10%)			

Median age: 59 years old

### Exosomes identification and characterization

**Fig. 1:**
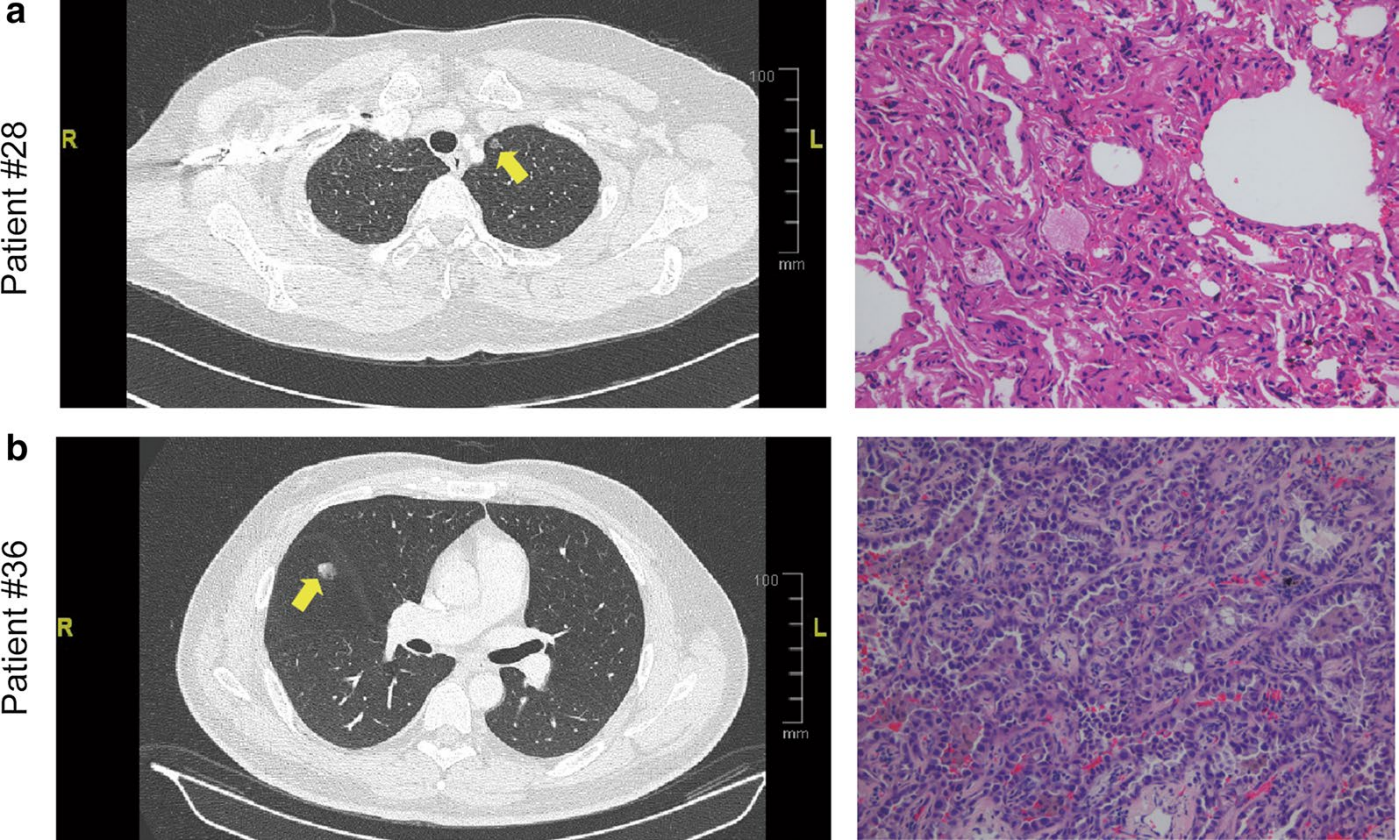
Imaging and pathology in patients with pulmonary nodules. **a** CT image of a representative patient with benign PN #28 who was diagnosed with focal fibrosis with inflammatory cell infiltration. Corresponding paraffin-embedded tissue was processed for H&E staining (× 200), **b** CT image of a representative patient with malignant PN #36 who was diagnosed with lung adenocarcinoma (acinar predominant), Corresponding paraffin-embedded tissue was processed for H&E staining (× 200)

**Fig. 2:**
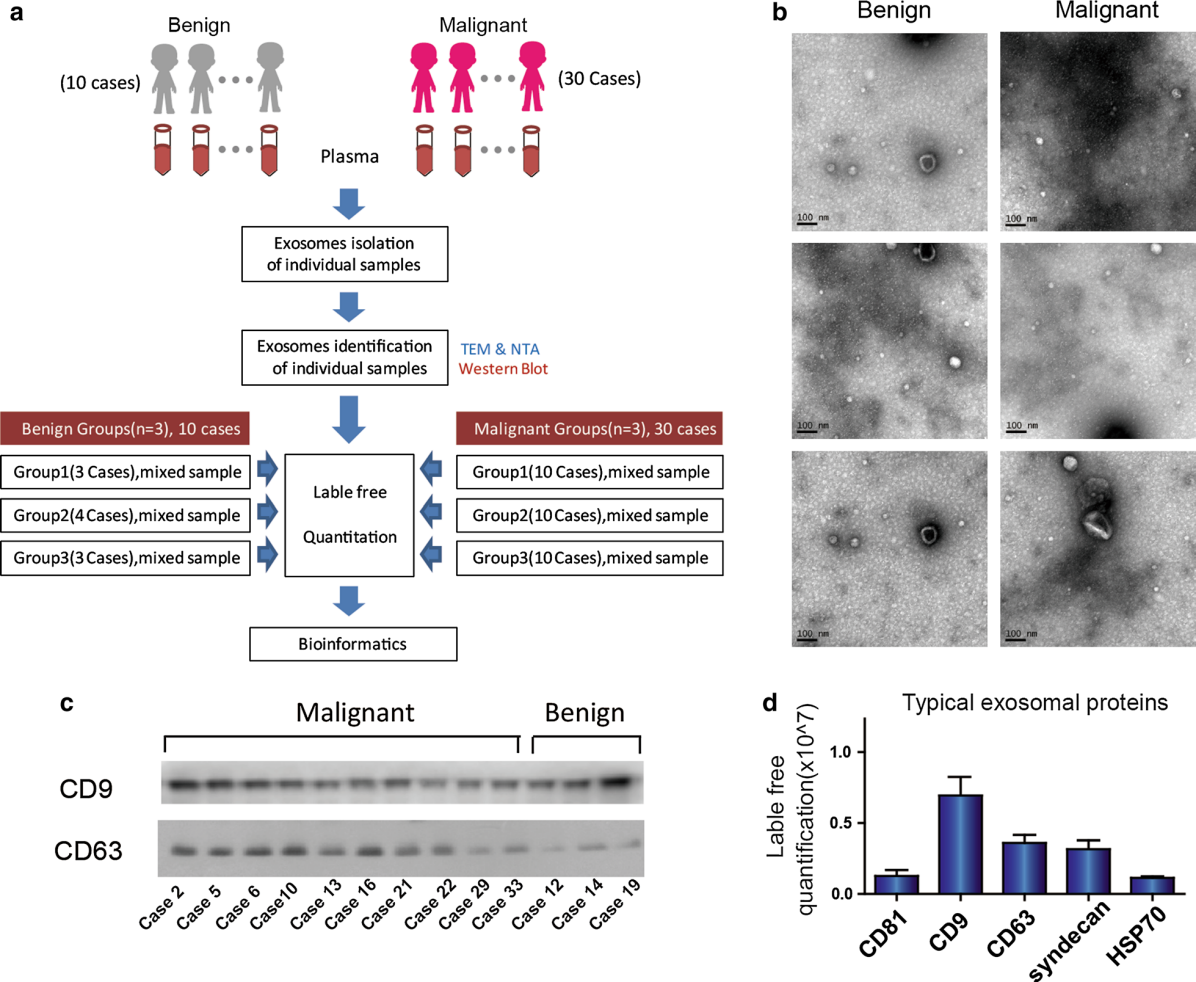
Exosomes extraction, identification and characterization. **a** Study design of present research. Samples were divided into 6 parts randomly before label-free mass spectrometry analyses: group1, group 2, group 3 for benign PNs and group1, group 2, group 3 for malignant PNs, each group including 3, 4, 3, 10, 10, 10 samples respectively. **b** Electron microscopy images of plasma exosomes, Bar = 100 nm. **c** CD9 and CD63 were detected in exsomes from 10 malignant PNs samples and 3 benign PNs samples by Western blotting. **d** Typical exosome proteins CD9, CD63, CD81, Syndecan and HSP70 were validated by mass spectrometry analyses

Representative CT images and pathological images of benign and malignant PNs patients are shown in Fig. 1a and b, respectively. We aimed to isolate exosome preparations from the plasma of patients with benign or malignant PNs. The study design was shown in Fig. 2a. The characterization of PNs from exosomes was subjected to several control analyses. Transmission electron microscopy was employed to characterize the quality of the vesicles. As shown in Fig. 2b, exosomes from patients with PNs had a visible circle-shape morphology in electron micrographs with a diameter of approximately 100 nm; the size of exosomes from patients with benign or malignant PNs was similar. We did NTA analysis to detect the size of exosome samples (Additional file 1: Figure S2). In addition, the typical exosomal markers CD9 and CD63 were detected by Western blot (Fig. 2c). In agreement with Fig. 2c, CD81, syndecan and HSP70 as well as CD9 and CD63 were present in the exosomes as detected by MS (Fig. 2d), confirming these vesicles as exosomes.

### Comparison of the proteomes of malignant/benign PNs plasma exosomes

**Fig. 3:**
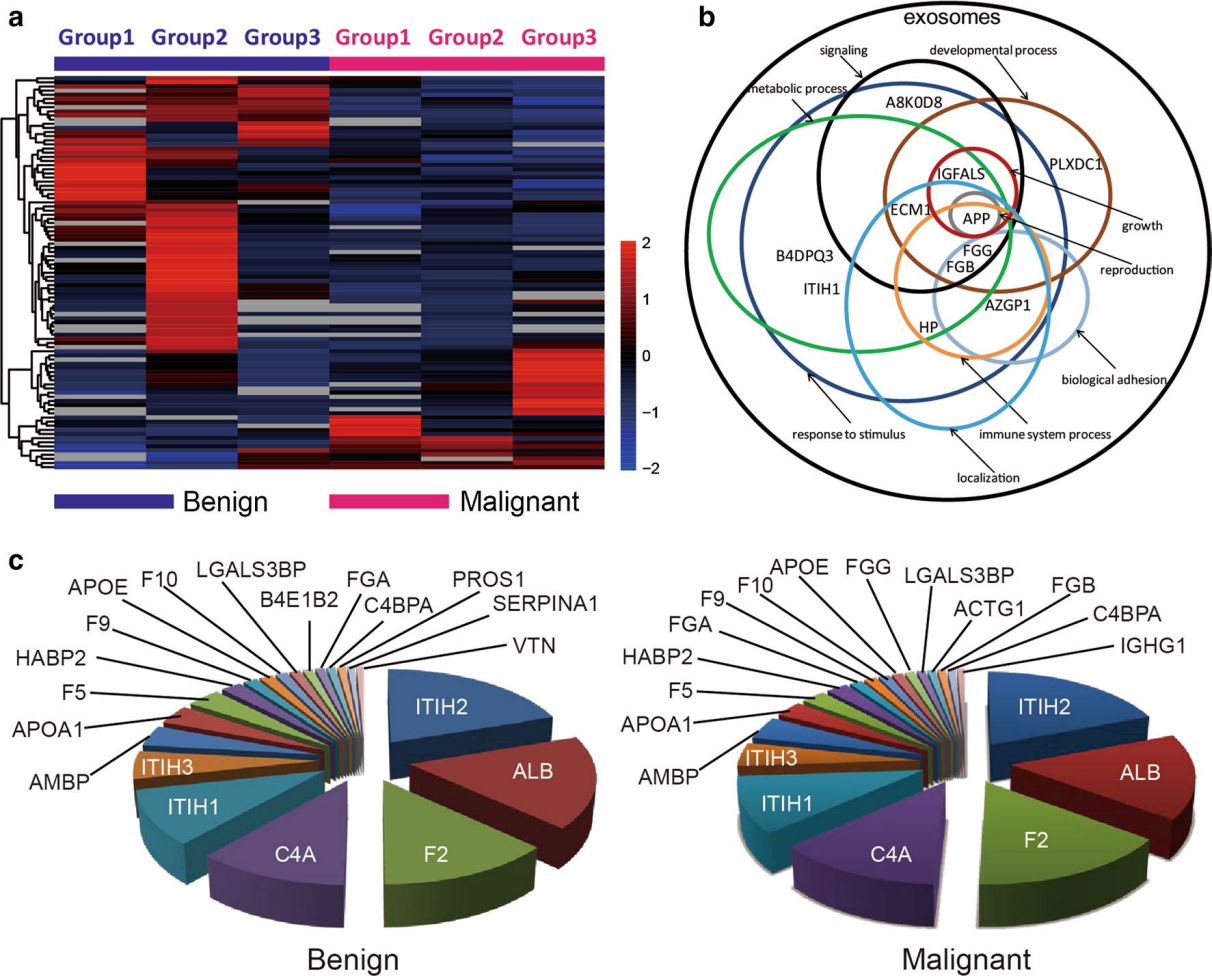
**a** The benign and malignant PNs cases were divided into 3 groups, respectively. Unsupervised hierarchical clustering was performed of proteins detected in 6 groups. Dark red means relatively higher expression, dark blue means relatively lower expression and gray indicates missing abundance values. **b** Venn diagram showing the overlap among functional clusters obtained according to Gene Ontology Data Base (level 2). **c** Pie diagrams of top 20 abundant proteins in benign and malignant samples as measured by mass spectrometry analyses

In the present study, we detected 736 proteins in the exosomes of patients with PNs by label-free analysis. The protein identification data were shown in Additional file 1: Table S2. Of these, 395 proteins were detected in both benign and malignant PNs. Through data analysis, we found that there were significant differences in the expression levels of 12 proteins in the benign and malignant PNs groups (multiplying multiples of 1.5 times and p < 0.05). Compared to the benign PNs group, FGG and FGB were significantly higher in the malignant PNs group, while IGFALS, PLXDC1, ITIH1, APP, C16orf46, AZGP1, B4DPQ3 (protein ID), A8K0D8 (protein ID), HP, and ECM1 were of significantly lower expression in the malignant PNs group. In addition, there were 21 other proteins detected only in the benign or malignant PNs group. NAPA, FLOT2, TPM1, NDRG4, ARPC5, DLD, MBL2, PSMA4, VDAC2 were only detected in the malignant PNs group, while Q59FP5 (protein ID), FBN1, CSF1, CSPG3, KIF5B-ALK, IGKV2D-28, LAMB1, CD81, VASN, CFHR4, IL7R, HLA-B were only detected in the benign PNs group. Differential protein expression is shown in Table 2. Volcano plot of present mass spectrometry was shown in Additional file 1: Figure S3. Hierarchical cluster analysis was applied to group benign and malignant PNs exosomes based on their LFQ intensity profiles. As shown in Fig. 3a, clustering the 395 proteins could perfectly separate the benign and malignant PNs exosomes. According to the GO database, these differentially enriched proteins were clustered into nine functional groups. A Venn diagram was constructed to clarify the overlap among these clusters (Fig. 3b), and the clusters are as follows: growth, reproduction, developmental processes, metabolic process, biological adhesion, immune system processes, signaling, response to stimulus and localization. From the present study, the top 20 most abundant proteins in plasma exosomes from patients with benign or malignant PNs are shown in Fig. 3c and show a high qualitative overlap (60%, 12 of 20 proteins) (data were listed in Additional file 1: Table S3 and Table S4). To preliminary validate the result of present study, GFALS, FGB and CD63 were detected by Western blotting (Additional file 1: Figure S4).

**Table 2:** Differentially expressed proteins between malignant PNs group and benign PNs group

Protein symbol	p value	Fold (malignant/benign)	Peptide	Unique peptides
IGFALS	0.00053096	0.318426426	13	13
PLXDC1	0.00193538	0.389271884	10	10
ITIH1	0.012967236	0.272101082	13	1
APP	0.018235495	0.386848501	11	11
FGB	0.003852587	1.656288542	37	37
C16orf46	0.006916273	0.651005857	1	1
AZGP1	0.013813029	0.610964507	14	14
FGG	0.019121291	1.55572236	31	4
B4DPQ3	0.019588885	0.633906834	15	15
A8K0D8	0.022714839	0.524011735	16	16
ECM1	0.030964495	0.617536089	8	8
HP	0.041580346	0.610498412	27	12
NAPA	▲	▲	2	2
FLOT2	▲	▲	2	2
TPM1	▲	▲	15	1
NDRG4	▲	▲	1	1
ARPC5	▲	▲	1	1
DLD	▲	▲	2	2
MBL2	▲	▲	1	1
PSMA4	▲	▲	2	2
VDAC2	▲	▲	2	2
Q59FP5	*	*	6	4
FBN1	*	*	11	11
CSF1	*	*	2	1
CSPG3	*	*	3	3
KIF5B-ALK	*	*	1	1
IGKV2D-28	*	*	3	1
LAMB1	*	*	6	6
CD81	*	*	2	2
VASN	*	*	1	1
CFHR4	*	*	2	1
IL7R	*	*	5	5
HLA-B	*	*	12	0

Asterisk “*” represents the detected protein in benign PNs group; Solid triangle “▲” represents the detected protein in malignant PNs group

### KEGG and gene ontology enrichment analyses of plasma exosome proteome

**Fig. 4:**
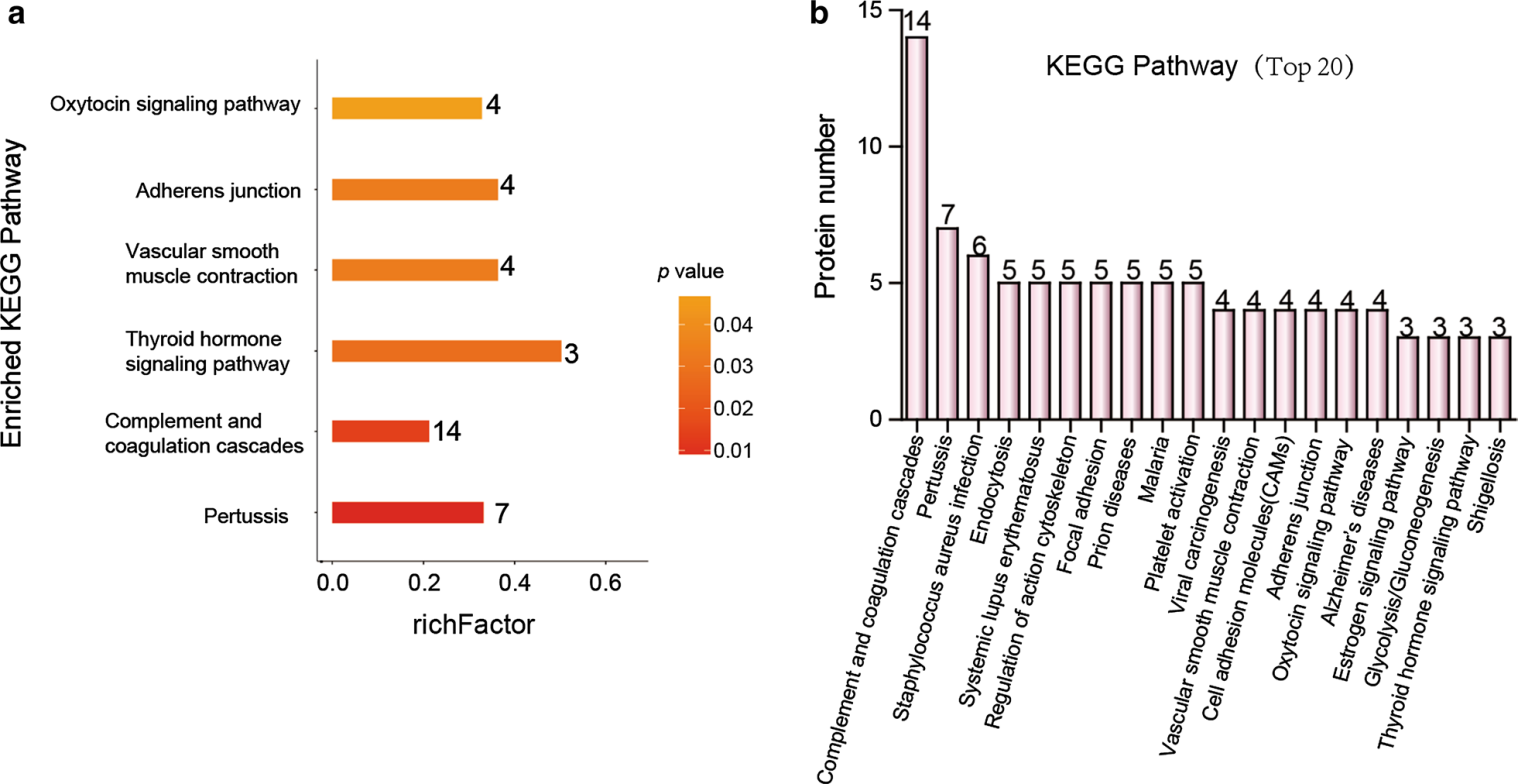
KEGG pathway enrichment was analyzed. **a** The ordinate represents the rich factor, and the abscissa represents the enriched KEGG pathways, **b** top 20 enriched KEGG pathways

Proteins were significantly altered between patients with malignant and benign PNs. Plasma exosomes were investigated to identify proteins of particular interest for distinguishing malignant and benign PNs. Enriched KEGG pathways were conducted to analyze the plasma exosome proteome. The results showed these significant pathways: oxytocin signaling, adherens junction, vascular smooth muscle contraction, thyroid hormone signaling, complement and coagulation cascade and pertussis (p value < 0.05) (Fig. 4a). The top 20 enriched KEGG pathways were illustrated in Fig. 4b. Among them, the most enriched pathway was the complement and coagulation cascade pathway.

**Fig. 5:**
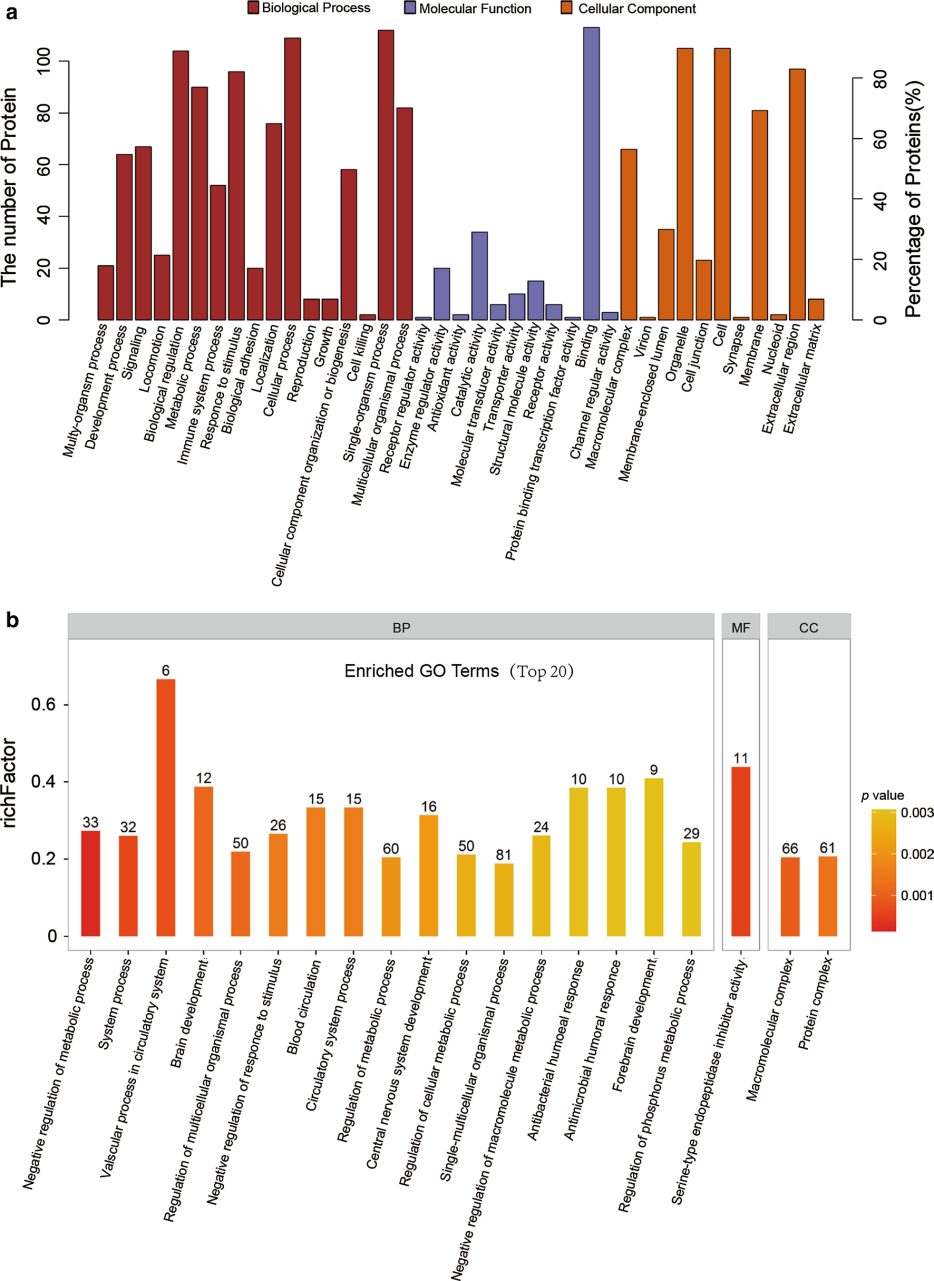
Histogram of enriched GO terms. **a** Gene oncology (GO) enrichment analysis of detected proteins, **b** top 20 enriched GO terms. The ordinate represents the enriched GO terms, and the abscissa represents the rich factor

**Fig. 6:**
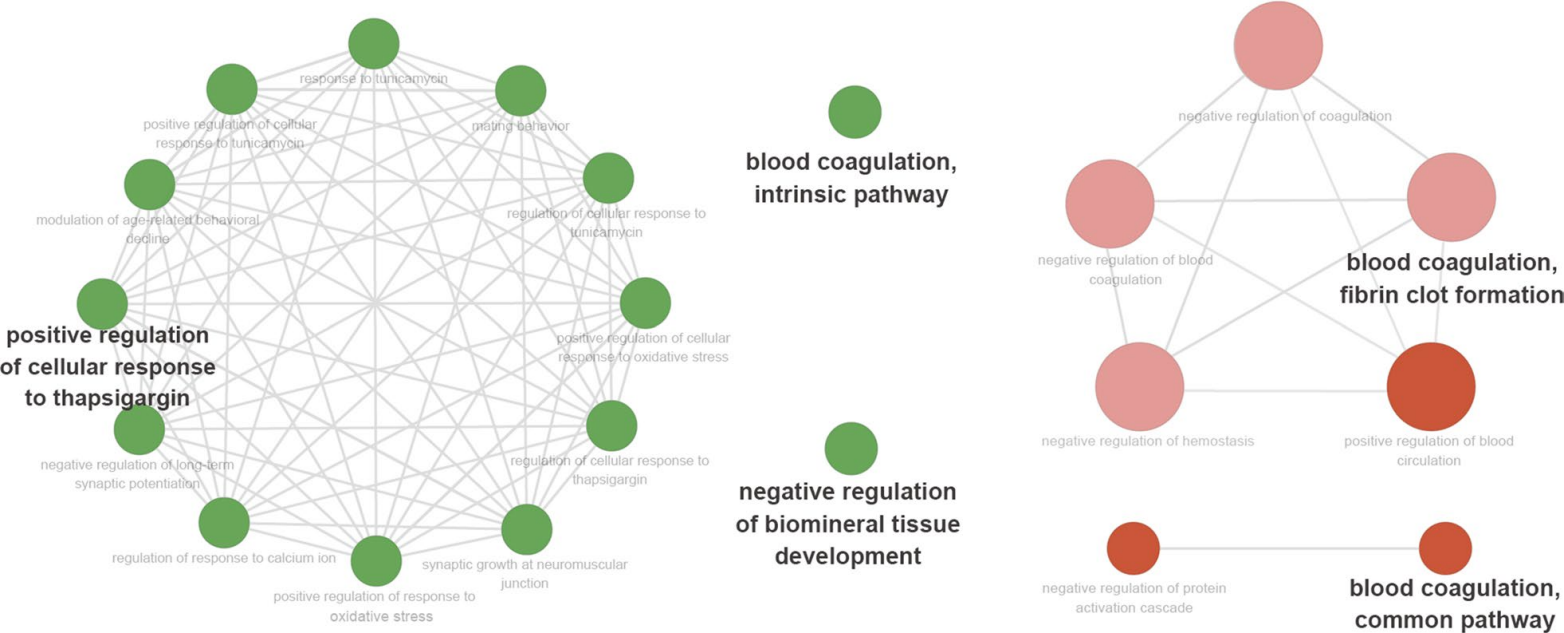
Protein GO enrichment analysis using the Cytoscape plugin ‘ClueGO’. In the network, only significantly enriched categories (p value < 0.05) are shown. The node color represents different groups of samples. Green represents the result from benign samples, red represents the result from malignant samples. The edges of the resulting ClueGO network are based on kappa statistics and reflect the relationships between the GO Biological Process (network nodes) based on the similarity of their associated proteins. The GO level was defined as level0–level3

Then, we classified these proteins according to their gene ontology. Proteins were analyzed for aspects of biological processes, molecular functions and cellular components (Fig. 5a). Furthermore, the top 20 enriched GO terms were illustrated in Fig. 5b with p value information, and the most significant term was the negative regulation of metabolic processes. Upon further research on significant upregulation in exosomes from patients with benign and malignant PNs (1.5 times and p < 0.05), the Cytoscape plugin ‘ClueGO’ was applied (Fig. 6). Significantly upregulated proteins in the benign group presented the biological function of positive regulation of cellular response to thapsigargin, negative regulation of biomineral tissue development and blood coagulation, intrinsic pathway, while the malignant group showed the biological functions of blood coagulation and fibrin clot formation.

### STRING interaction analysis of the significantly up/downregulated proteins

**Fig. 7:**
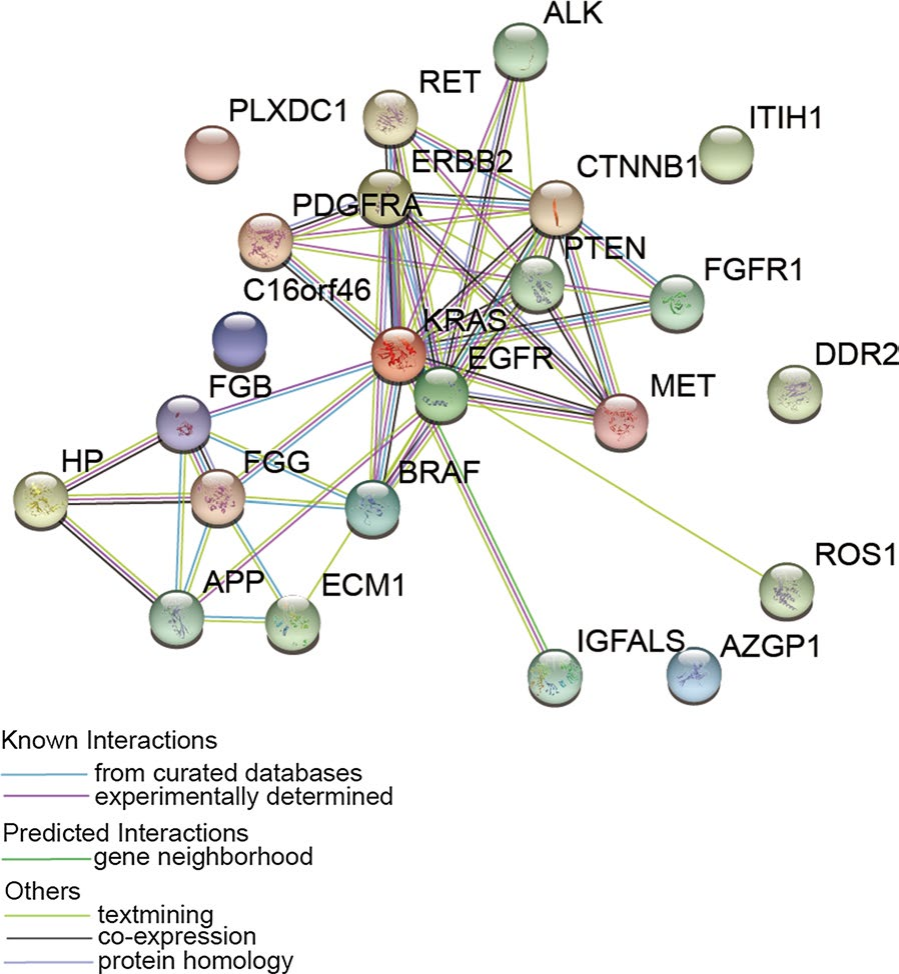
STRING protein interaction analysis of proteins significantly up/down-regulated in plasma exosomes from patients with malignant PNs. ALK, EGFR, FGFR1, ROS, RET, KRAS, BRAF, MET, PTEN, PI3KCA, DDR2, HER2, CTNNB1, and PDGFRA were also indicated

Twelve significantly up/downregulated (1.5 times and p < 0.05) proteins were included in a STRING network (Version: 10.5 https://string-db.org/), together with some proteins: ALK, EGFR, FGFR1, ROS, RET, KRAS, BRAF, MET, PTEN, PI3KCA, DDR2, HER2, CTNNB1 and PDGFRA. As illustrated in Fig. 7, protein–protein interactions were marked; these proteins may have a specific role in exosome release from malignant PNs.

## Discussion

PNs always present as focal lesions, but not all PNs are malignant. Most PNs also present similar clinical and imaging features, and they do not all require aggressive treatment. Therefore, it is very important for making a treatment plan to differentiate benign from malignant PNs. Despite the fact that malignant PNs can be detected at an earlier stage by low-dose spiral computed tomography (LDCT) [15], the distinction between benign and malignant PNs remains very difficult to determine. In addition, advanced imaging techniques require complex procedures with high costs, false positives and other consequences such as high rates of repeated CT scans for further assessment. Lung biopsy was considered the primary reference method for diagnosis of malignant PNs, but it has many limitations such as invasiveness, sampling error, and inter-observer variability [16]. However, compared with imaging and histological detection methods, blood detection has the advantages of low cost and convenience and is more suitable for dynamic detection.

The term exosome was initially coined by Johnstone et al. [17]. In recent years, the significant detection function of molecular biomarkers in exosomes has been discovered, since glypican-1 was found to identify cancer exosomes and could detect early pancreatic cancer [18]. Other biological molecules such as microRNA [19–21] and lncRNA [22, 23] can be specific markers of cancer exosomes and detect early stages of specific cancers using plasma or serum from patients. Exosomes have various names such as oncosomes, tolerosomes, argosomes, etc. that reflect that they are involved in a wide variety of physiological and pathological tumor events. In this way, exosomes can play a key role in preoperative differentiation of benign versus malignant PNs. To elucidate which proteins could be of relevance among the exosomes’ cargo as they can affect the potential of PNs, we performed a proteomic analysis from exosomes derived from patients with benign and malignant PNs. In the present study, we classified proteins according to their gene ontology. The most common cellular component (level 6) was extracellular vesicular exosomes that corroborated the source of those proteins. The main biological processes recognized as the major purpose of the exosomes were the regulation of cellular processes and cell communication. Furthermore, the predominant molecular functions featured proteins in this group known to be implicated in that binding. Otherwise, proteins detected in exosomes were analyzed using KEGG database. The pathways with more proteins implicated included focal adhesion, CAMs, proteoglycans in cancer, PI3K-Akt signaling pathway et al., that might be involved in tumor genesis and metastasis.

Our results are based on proteomic analysis of more than 30 malignant PNs patients and 10 benign PNs samples, which are the source of several pathological biomarkers and have clinical utility. To our knowledge, this is the first attempt to find biomarkers for the identification of benign and malignant PNs from the perspective of exosomal proteomics. Several of these proteins have been identified previously in exosomes and some are novel. Among them, FGB and FGG were significantly up-regulated in patients with malignant PNs (fold change > 1.5 times). FGB is cleaved by the protease thrombin to yield monomers that polymerize to form an insoluble fibrin matrix and is usually associated with coagulation disorders [24], diseases of the circulatory system [25], infection [26], immune response [27] etc. Recently, many unexpected discoveries shed a new light on FGB. The expression of FGB increased with stage (p < 0.001) in bladder cancer and is a potential marker to characterize and diagnose bladder cancer [28]. Otherwise, using iTRAQ and mass spectrometry analysis, researchers found that FGB was significantly up-regulated in laryngeal carcinoma tissues [29]. FGG was significantly up-regulated at the mRNA level in hepatocellular carcinoma (HCC) compared to non-cancerous adjacent tissues and may serve as a useful predictor of clinical progression of HCC patients [30]. Proteomics analysis of urine reveals that FGG can serve as a candidate diagnostic biomarker for prostate cancer [31]. ITIH and IGFALS were significantly up-regulated in patients with benign PNs (fold change > 1.5 times). ITIH molecules, which consist of five members (ITIH1-5), have been shown to play a particularly essential role in inflammation and carcinogenesis, showing frequent down-regulation that may be associated with initiation and/or progression of multiple human solid tumors [32]. The down-regulated IGFALS was associated with etiology and progression of breast cancer, prostate cancer and testicular germ cell tumors [33–35], and it was identified to function as a tumor suppressor in liver cancer [36]. In addition, the expression level of IGFALS was linked to coronary heart disease or stroke among postmenopausal women [37].

Based on previous research, several biomarkers have been described in exosomes of lung cancer (compared to normal tissue samples), including several miRNAs and EpCAM from plasma exosomes [38] and LRG1 from urine exosomes [39]. Previous studies in patients with non-small cell lung cancer have shown that proteins commonly used to screen tumor biomarkers can be used as exosome biomarkers of non-small cell lung cancer, such as EGFR, CD151, CD171, tetraspanin 8 [40], and NY-ESO-1 [41]. Few reports described the use of exosomal proteins as PNs diagnosis biomarkers. One of the advantages of using PNs plasma biomarkers versus lung tissues is that plasma exosomes provide a global profile of the tissue compared to lung biopsies due to the heterogeneity of lung tissue. It is worth noting that a panel of biomarkers, rather than a single molecule, will be required due to the complexity of lung cancer. This study has some potential limitations. Some of the differential proteins that we screened and analyzed still need further validation in clinical practice in the future. In addition, further studies with a larger cohort of patients and serial blood samples are needed to validate our findings.

## Additional file

**Additional file 1.** Additional figures and tables.
