# Supplementary material for: Proteomic analysis of plasma exosomes to differentiate malignant from benign pulmonary nodules
Source: Clin Proteomics. 2019 Feb 2;16:5. doi: 10.1186/s12014-019-9225-5 (PMC6359787; doi:10.1186/s12014-019-9225-5)
Supplement: Supplementary file 1 — Additional file 1. Additional figures and tables. [file 12014_2019_9225_MOESM1_ESM.docx]

**Additional file 1**


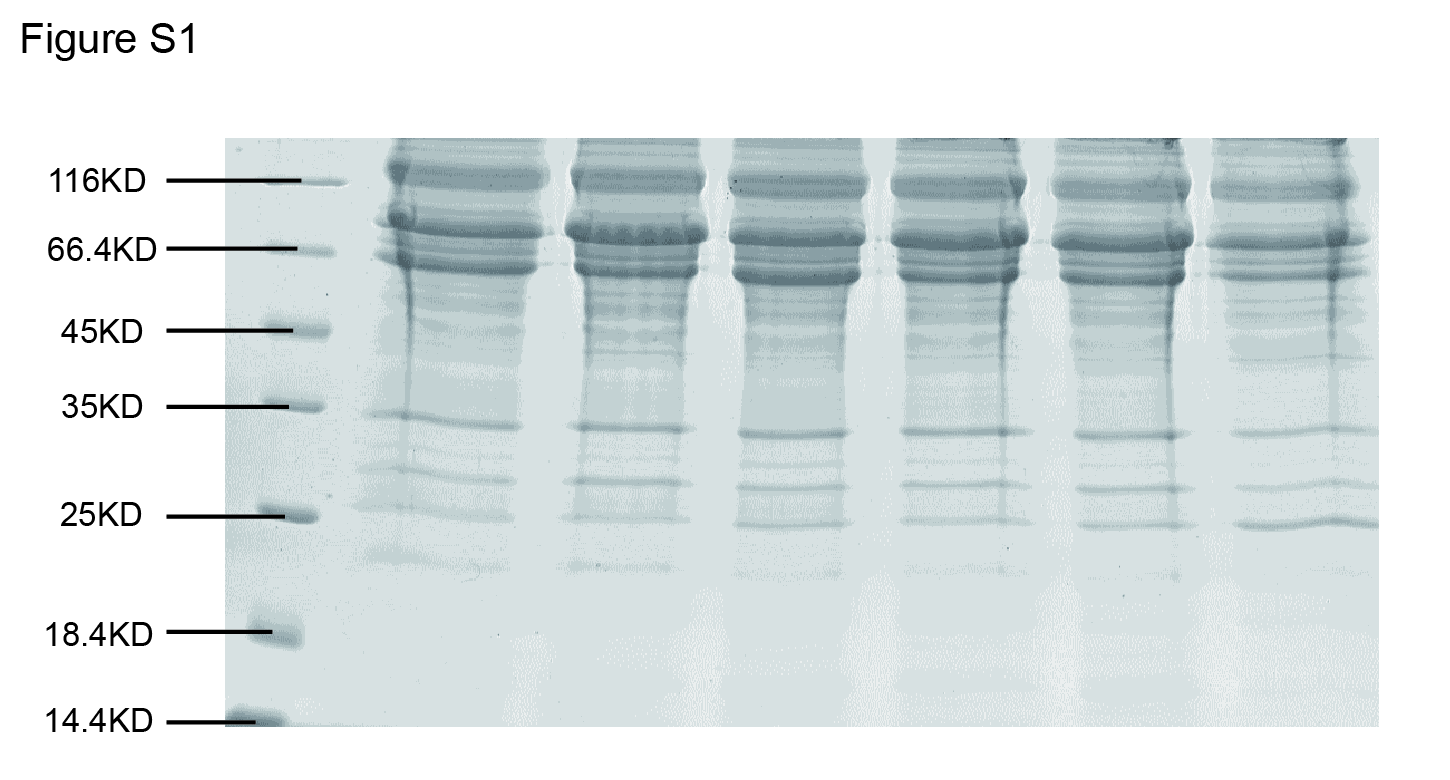


Figure S1. Proteins from each group were used for SDS-PAGE analysis. Coomassie blue staining was applied for detecting proteins for six groups (from left to right: group 1, group2, group3 of benign PNs and group 1, group2, group3 of malignant PNs).


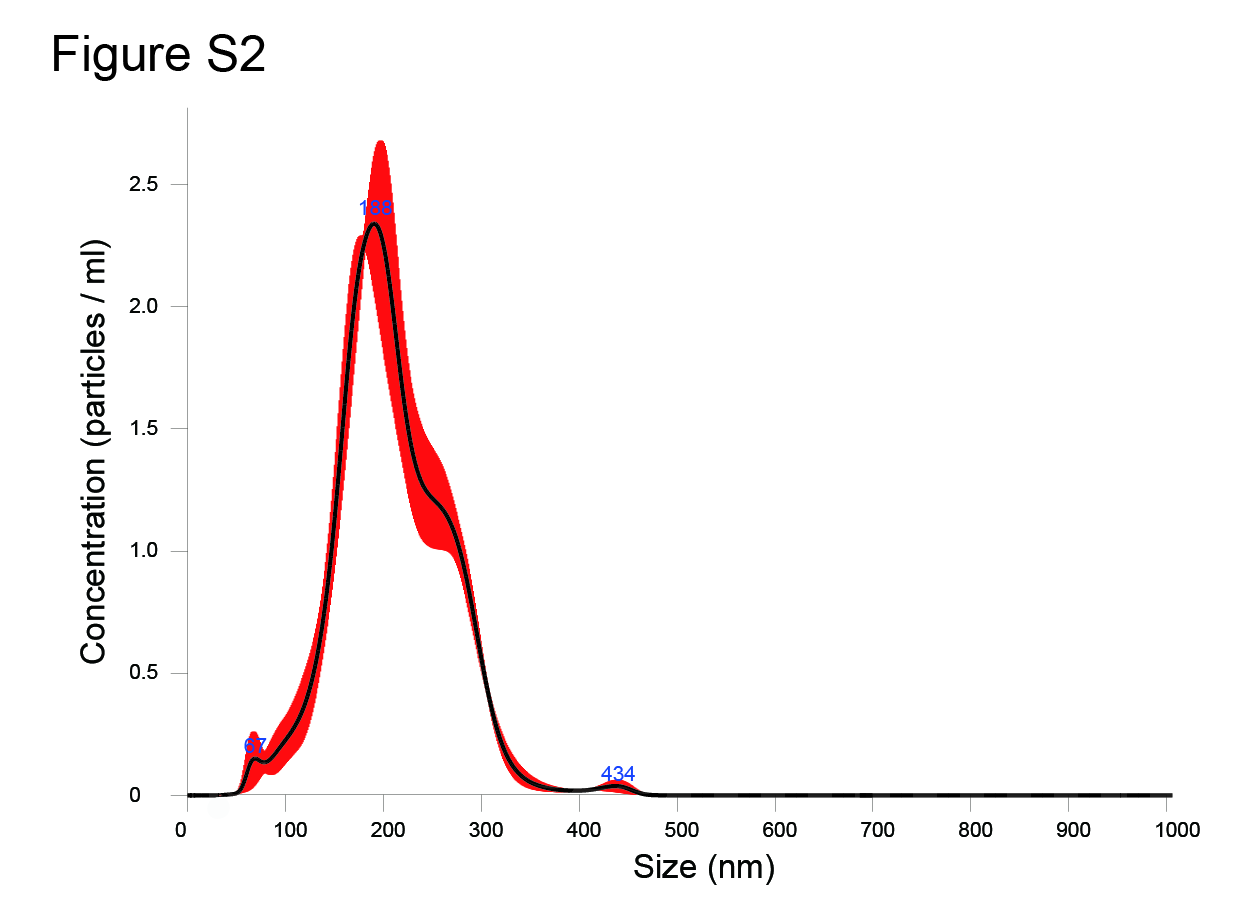


Figure S2. Sizes distribution of the plasma exosomes. Nanoparticle Tracking Analysis (NTA) of exosome samples from the plasma of PN patients.


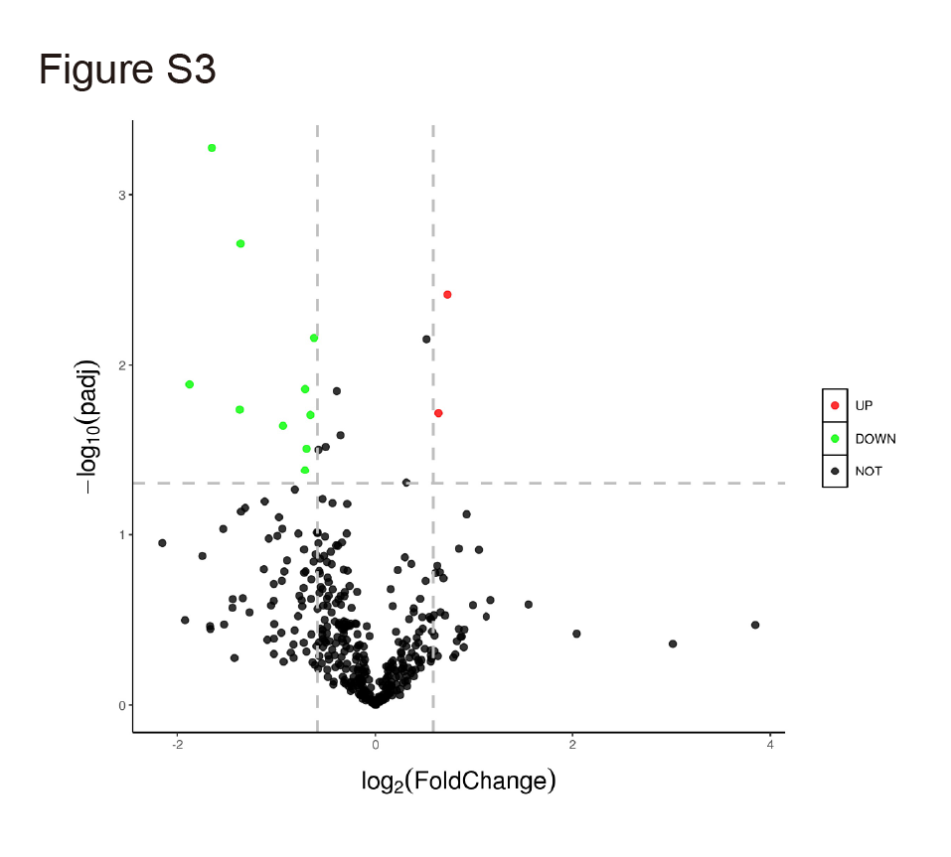


Figure S3. Volcano plot of present Mass Spectrometry. Red bolt means the protein fold changs of malignant/benign was > 1.5 times with p value < 0.05, and green bolt means the protein fold changs of benign/malignant was > 1.5 times with p value < 0.05.


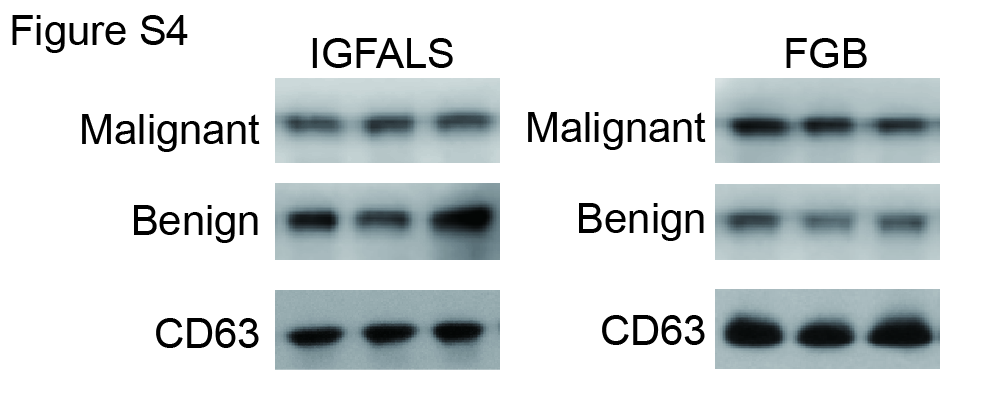


Figure S4. Western Blotting of GFALS, FGB and CD63.

**Table S1. Protein concentration of exosome samples.**

| M1 | M2 | M3 | M4 | M5 | M6 | M7 | M8 | M9 | M10 |
| --- | --- | --- | --- | --- | --- | --- | --- | --- | --- |
| 2.114195 | **2.18261** | **1.931755** | **1.794925** | **1.703705** | **1.794925** | **1.658095** | **1.749315** | **2.114195** | **1.45285** |
| M11 | **M12** | **M13** | **M14** | **M15** | **M16** | **M17** | **M18** | **M19** | **M20** |
| 1.749315 | **1.49846** | **1.72651** | **1.658095** | **2.342245** | **1.95456** | **2.022975** | **2.27383** | **1.977365** | **2.163595** |
| M21 | **M22** | **M23** | **M24** | **M25** | **M26** | **M27** | **M28** | **M29** | **M30** |
| 1.794925 | **1.977365** | **1.886145** | **1.95456** | **1.49846** | **1.81773** | **1.6809** | **2.00017** | **2.00017** | **1.86334** |
| B1 | **B2** | **B3** | **B4** | **B5** | **B6** | **B7** | **B8** | **B9** | **B10** |
| 1.703705 | **1.019555** | **1.384435** | **1.384435** | **1.45285** | **1.521265** | **1.36163** | **1.72651** | **1.293215** | **1.703705** |

M:malignant,B:benign,unit: ug/ul

**Table S2. Detected proteins of malignant and benign PNs group.**

| **Protein symbol** | ***p*_Value** | **Fold（M/B)** | |  | | **Protein symbol** | | | ***p*_Value** | | **Fold（M/B)** | | | |
| --- | --- | --- | --- | --- | --- | --- | --- | --- | --- | --- | --- | --- | --- | --- |
| ESYT2 | NA | NA |  | | STX7 | | | NA | | | | NA |  |  |
| IGLC7 | NA | NA |  | | ARPC5 | | | ▲ | | | | ▲ |  |  |
| SSC5D | 0.2164580266 | 0.8794647945 |  | | PLXNB1 | | | 0.6783809688 | | | | 1.0886382242 |  |  |
| SIGLEC16 | 0.3764660475 | 0.5175329639 |  | | TGFB1I1 | | | NA | | | | NA |  |  |
| ANXA2P2 | NA | NA |  | | AKR7A2 | | | NA | | | | NA |  |  |
| PDLIM1 | 0.8733551860 | 1.1875861725 |  | | ASNA1 | | | NA | | | | NA |  |  |
| SNAP23 | NA | NA |  | | ACTN4 | | | NA | | | | NA |  |  |
| MASP2 | 0.3332020447 | 0.8411975848 |  | | CALU | | | 0.8634731581 | | | | 0.9293188775 |  |  |
| RAB27B | 0.2357344468 | 0.3953270323 |  | | CD5L | | | 0.5802574056 | | | | 1.1321681376 |  |  |
| PGRMC1 | NA | NA |  | | SUSD5 | | | 0.3356187764 | | | | 0.7723550234 |  |  |
| CLIC1 | NA | NA |  | | KIF5BNAALK | | | * | | | | * |  |  |
| BPGFNA1 | NA | NA |  | | NRP2 | | | 0.2620740678 | | | | 0.7154860443 |  |  |
| GOLIM4 | NA | NA |  | | JAK2 | | | NA | | | | NA |  |  |
| PODXL | 0.6848226661 | 0.8583392292 |  | | Q59ER5 | | | NA | | | | NA |  |  |
| CSPG3 variant protein | * | * |  | | ROCK2 | | | NA | | | | NA |  |  |
| ADAM10 | NA | NA |  | | SH3BGRL | | | NA | | | | NA |  |  |
| NRP1 | 0.3222485772 | 0.8018096112 |  | | CS | | | NA | | | | NA |  |  |
| PSMA7 | NA | NA |  | | STX11 | | | NA | | | | NA |  |  |
| MYL12A | NA | NA |  | | SKAP2 | | | 0.5556245522 | | | | 0.5260001463 |  |  |
| FYB | NA | NA |  | | FCN3 | | | NA | | | | NA |  |  |
| ARPC1B | NA | NA |  | | ATRN | | | NA | | | | NA |  |  |
| ARPC2 | NA | NA |  | | ARL6IP5 | | | NA | | | | NA |  |  |
| SPTLC2 | 0.6975995252 | 0.8896328792 |  | | CA1 | | | NA | | | | NA |  |  |
| ATP5H | NA | NA |  | | CA2 | | | NA | | | | NA |  |  |
| ECM2 | 0.2857281774 | 0.7352135759 |  | | SERPINC1 | | | 0.1301059759 | | | | 0.6589697352 |  |  |
| ENDOD1 | NA | NA |  | | SERPINA1 | | | 0.0976606471 | | | | 0.6678461491 |  |  |
| CDK13 | NA | NA |  | | B3KS79 | | | 0.0257829867 | | | | 0.7834660622 |  |  |
| CLSTN1 | 0.2513909904 | 0.7577850304 |  | | Q59EP2 | | | 0.2055819915 | | | | 0.6050732063 |  |  |
| UTS2 | 0.1414795592 | 0.5385938648 |  | | A2M | | | 0.2626614885 | | | | 1.1300236407 |  |  |
| APOM | 0.1688873911 | 0.6783252676 |  | | C3 | | | 0.9336027332 | | | | 0.9934392602 |  |  |
| SDPR | 0.7950716222 | 0.8901442809 |  | | C5 | | | 0.1645167186 | | | | 0.5283830392 |  |  |
| G6B | NA | NA |  | | KNG1 | | | 0.0543713048 | | | | 0.5691749620 |  |  |
| LDHA | NA | NA |  | | TGFB1 | | | NA | | | | NA |  |  |
| CYB5R3 | NA | NA |  | | JCHAIN | | | 0.6346855224 | | | | 1.0906434161 |  |  |
| SOD1 | NA | NA |  | | P01608 | | | NA | | | | NA |  |  |
| CP | 0.1157469830 | 0.7586228101 |  | | P01617 | | | * | | | | * |  |  |
| F8 | 0.2426057571 | 0.5973689076 |  | | IGLV3NA19 | | | NA | | | | NA |  |  |
| F13A1 | 0.5626656883 | 1.3611525527 |  | | IGHG4 | | | 0.5091762129 | | | | 0.7522477159 |  |  |
| Q8N7G1 | NA | NA |  | | HBD | | | 0.4936862778 | | | | 1.2915029726 |  |  |
| PGK1 | NA | NA |  | | KRT14 | | | 0.0314283771 | | | | 0.6711100896 |  |  |
| F2 | 0.1358720881 | 1.2298019702 |  | | KRT6A | | | NA | | | | NA |  |  |
| HP | 0.0415803463 | 0.6104984120 |  | | SPTA1 | | | NA | | | | NA |  |  |
| HPR | 0.8017767138 | 1.0735602458 |  | | APOA1 | | | 0.6365025207 | | | | 0.8870452285 |  |  |
| F9 | 0.4532064484 | 0.9211753731 |  | | APOE | | | 0.4656570571 | | | | 0.8356158475 |  |  |
| F10 | 0.7884457908 | 0.9681913150 |  | | APOA2 | | | 0.2027816394 | | | | 0.6822545661 |  |  |
| PLG | 0.4360972909 | 1.2111213491 |  | | APOC1 | | | 0.2290215916 | | | | 0.7841012644 |  |  |
| APOC3 | 0.6771417957 | 1.1795900939 |  | | KLKB1 | | | NA | | | | NA |  |  |
| FGA | 0.0070442591 | 1.4292554353 |  | | C4BPA | | | 0.8703580857 | | | | 0.9643427309 |  |  |
| FGB | 0.0038525872 | 1.6562885415 |  | | VTN | | | 0.5075723329 | | | | 0.9203804013 |  |  |
| FGG | 0.0191212908 | 1.5557223596 |  | | CAT | | | 0.7619481510 | | | | 1.0886505615 |  |  |
| GYPA | NA | NA |  | | B4DPQ3 | | | 0.0195888846 | | | | 0.6339068342 |  |  |
| SLC4A1 | 0.7738068533 | 1.2474174225 |  | | ALDOA | | | 0.8174187728 | | | | 1.1373380710 |  |  |
| CRP | 0.3388794129 | 14.395457928 |  | | SOD2 | | | 0.9910805997 | | | | 1.0063669379 |  |  |
| APCS | 0.4850840186 | 0.8047685071 |  | | LCAT | | | NA | | | | NA |  |  |
| C1QA | 0.1596556936 | 0.4583639316 |  | | A1BG | | | 0.2169413444 | | | | 0.8069141703 |  |  |
| C1QB | 0.2686986787 | 0.3678687132 |  | | KRT1 | | | 0.7900246046 | | | | 1.0976186767 |  |  |
| C1QC | 0.4300652281 | 0.6056778618 |  | | VWF | | | 0.5636890792 | | | | 1.1188651977 |  |  |
| C9 | 0.7256605800 | 0.9134157812 |  | | GAPDH | | | 0.3553918847 | | | | 1.3929541256 |  |  |
| APOH | 0.9052689760 | 1.0264517214 |  | | CAPNS1 | | | NA | | | | NA |  |  |
| LRG1 | 0.1017462688 | 0.5039373077 |  | | HSPB1 | | | NA | | | | NA |  |  |
| RBP4 | 0.7407183637 | 0.8616434219 |  | | GNAI2 | | | 0.5690168436 | | | | 0.6875744558 |  |  |
| AMBP | 0.5487488672 | 1.1318187975 |  | | CHGB | | | NA | | | | NA |  |  |
| ORM1 | 0.9561696797 | 1.0120975488 |  | | APP | | | 0.0182354947 | | | | 0.3868485008 |  |  |
| B7Z8Q2 | 0.4537936340 | 0.8154706589 |  | | APOD | | | 0.7288115852 | | | | 1.0493646080 |  |  |
| A6XGL1 | 0.7925734688 | 1.0804727197 |  | | ITGB3 | | | 0.9827666105 | | | | 1.0082954347 |  |  |
| ALB | 0.9332630181 | 0.9811460613 |  | | S100A8 | | | NA | | | | NA |  |  |
| PPBP | 0.8783320562 | 1.1243427441 |  | | Q59EI9 | | | NA | | | | NA |  |  |
| PF4 | 0.8740472141 | 0.9009156184 |  | | SERPINA5 | | | 0.0733215302 | | | | 0.3903705616 |  |  |
| TF | 0.4764800866 | 1.4729270023 |  | | SERPING1 | | | 0.0971484177 | | | | 0.6634884274 |  |  |
| HPX | 0.2194843444 | 0.6758202304 |  | | CFI | | | 0.5879214583 | | | | 1.2194739426 |  |  |
| F13B | NA | NA |  | | PFN1 | | | 0.8825731159 | | | | 0.9179811220 |  |  |
| SERPINA7 | 0.8338022820 | 0.9396663563 |  | | HSP90AA1 | | | 0.3402631448 | | | | 0.7740568037 |  |  |
| SERPIND1 | 0.6378692183 | 0.9400146988 |  | | LAMB1 | | | * | | | | * |  |  |
| ITGB1 | NA | NA |  | | LYN | | | NA | | | | NA |  |  |
| MYL3 | NA | NA |  | | THBS1 | | | 0.9258983999 | | | | 1.0264039715 |  |  |
| P06313 | 0.7445366623 | 0.9028919090 |  | | RHOC | | | NA | | | | NA |  |  |
| GSN | 0.9271855347 | 0.9646193383 |  | | SERPINA6 | | | 0.4209259761 | | | | 0.7250097725 |  |  |
| ATP5B | 0.3572176315 | 0.3149367470 |  | | HSP90AB1 | | | 0.5216903967 | | | | 0.8899656963 |  |  |
| S100A9 | NA | NA |  | | SOD3 | | | NA | | | | NA |  |  |
| S100A6 | NA | NA |  | | ITGA2B | | | 0.7552774143 | | | | 1.1575487577 |  |  |
| APOA4 | 0.2728193893 | 0.6659985415 |  | | LPA | | | 0.4145256418 | | | | 1.2434967430 |  |  |
| ENO1 | 0.9334330870 | 1.0555401447 |  | | PLEK | | | 0.7565961609 | | | | 0.7448538235 |  |  |
| LPL | NA | NA |  | | CD14 | | | 0.7040303664 | | | | 0.9081246308 |  |  |
| LDHB | 0.5153663203 | 1.5495968768 |  | | PTPRC | | | NA | | | | NA |  |  |
| PROS1 | 0.1258282944 | 0.7316553016 |  | | CFH | | | 0.0660023699 | | | | 0.8206875283 |  |  |
| P4HB | 0.1491466930 | 0.7362183110 |  | | FCGR3A | | | NA | | | | NA |  |  |
| CSF1R | 0.3525493708 | 0.8041317179 |  | | SERPINF2 | | | 0.2397364721 | | | | 0.7716940333 |  |  |
| C8A | 0.7642303854 | 0.8793339212 |  | | F7 | | | NA | | | | NA |  |  |
| C8B | 0.1636705927 | 0.6747940801 |  | | KRT16 | | | NA | | | | NA |  |  |
| GP1BA | 0.0301709029 | 0.7059761363 |  | | GSTP1 | | | NA | | | | NA |  |  |
| C8G | NA | NA |  | | SPARC | | | 0.4410228516 | | | | 0.5633015763 |  |  |
| CAPN1 | NA | NA |  | | CSF1 | | | * | | | | * |  |  |
| TUBB | 0.8075874842 | 0.8550528611 |  | | PDGFRB | | | NA | | | | NA |  |  |
| PRSS2 | 0.1120476032 | 0.2252293027 |  | | DLD | | | ▲ | | | | ▲ |  |  |
| C1S | 0.1110984123 | 0.7914146341 |  | | ACTN1 | | | 0.3959162738 | | | | 1.8308598630 |  |  |
| B7Z3K9 | 0.0759976319 | 1.8936934246 |  | | CDH1 | | | NA | | | | NA |  |  |
| SAA2 | NA | NA |  | | SRC | | | 0.5011674537 | | | | 0.4911519088 |  |  |
| Q59EJ3 | NA | NA |  | | GP1BB | | | 0.7178611810 | | | | 1.2492793479 |  |  |
| SRGN | NA | NA |  | | CCL5 | | | NA | | | | NA |  |  |
| SPP1 | 0.7314053860 | 1.1176829421 |  | | NCAM1 | | | 0.4333607835 | | | | 0.7571855998 |  |  |
| TXN | NA | NA |  | | Q59ED3 | | | NA | | | | NA |  |  |
| CTSA | NA | NA |  | | Q59FG9 | | | 0.8464780061 | | | | 1.0893523488 |  |  |
| C7 | NA | NA |  | | P13645 | | | 0.2970814224 | | | | 1.5058533876 |  |  |
| CHGA | 0.2707799298 | 1.3062677309 |  | | KRT5 | | | 0.3640262190 | | | | 0.5683199550 |  |  |
| PF4V1 | NA | NA |  | | PDIA4 | | | 0.9293283186 | | | | 0.9769593770 |  |  |
| HSPD1 | NA | NA |  | | C6 | | | NA | | | | NA |  |  |
| CLU | 0.1160603515 | 0.7690987392 |  | | PRG2 | | | 0.1644449316 | | | | 0.6126943507 |  |  |
| HSPA5 | 0.4604997150 | 0.8780918530 |  | | HLANAE | | | NA | | | | NA |  |  |
| LAMC1 | 0.0617837927 | 0.6912440203 |  | | COL11A2 | | | NA | | | | NA |  |  |
| HSPA8 | 0.3601665851 | 1.8626866463 |  | | SELL | | | 0.2294424710 | | | | 0.8049218434 |  |  |
| Q59GX2 | 0.4826887380 | 1.5120848373 |  | | CD99 | | | NA | | | | NA |  |  |
| B7Z5J8 | 0.3979111555 | 1.8148287911 |  | | B4DJQ5 | | | 0.9720565850 | | | | 0.9945547270 |  |  |
| Q59GM9 | NA | NA |  | | NID1 | | | 0.7297723992 | | | | 1.0300804986 |  |  |
| MBL2 | ▲ | ▲ |  | | PKM | | | 0.6197631162 | | | | 1.3248354510 |  |  |
| RALB | NA | NA |  | | HSP90B1 | | | 0.5657992305 | | | | 0.9196444545 |  |  |
| Q59FP5 | * | * |  | | GP9 | | | 0.2566296228 | | | | 2.9388400859 |  |  |
| CETP | 0.0985977230 | 0.5827731749 |  | | RAC1 | | | 0.7314354609 | | | | 0.8428507086 |  |  |
| F5 | 0.0141996898 | 0.7626028422 |  | | CSRP1 | | | NA | | | | NA |  |  |
| CPN1 | 0.0790653498 | 0.5091938932 |  | | FLNA | | | 0.5547486092 | | | | 1.4742842293 |  |  |
| CD44 | 0.3284492403 | 0.7750331758 |  | | CD9 | | | 0.8727215025 | | | | 1.0615612831 |  |  |
| SELP | NA | NA |  | | TNXB | | | 0.1605166217 | | | | 0.8004099369 |  |  |
| ACAN | 0.2284394719 | 0.7205146911 |  | | GPX3 | | | 0.3464468219 | | | | 0.7138202828 |  |  |
| ANK1 | NA | NA |  | | NME1NANME2 | | | NA | | | | NA |  |  |
| PECAM1 | 0.4138709470 | 0.4689840982 |  | | CPN2 | | | 0.3624654422 | | | | 0.8012897660 |  |  |
| EPB42 | NA | NA |  | | PROZ | | | 0.9266866792 | | | | 1.0217447136 |  |  |
| ATP2A2 | NA | NA |  | | P23083 | | | 0.5840408643 | | | | 0.7827152727 |  |  |
| UGT2B7 | NA | NA |  | | FBLN1 | | | 0.6227905348 | | | | 0.9105874221 |  |  |
| CD36 | 0.6133683330 | 1.2025942700 |  | | PTGS1 | | | NA | | | | NA |  |  |
| HSPA6 | 0.9762677172 | 0.9897628874 |  | | ITGA6 | | | 0.9378439510 | | | | 0.9355526269 |  |  |
| ITGA2 | NA | NA |  | | PPIB | | | 0.9510646772 | | | | 1.0355298062 |  |  |
| Q59EF6 | NA | NA |  | | PTPRG | | | 0.1054206408 | | | | 0.4741247743 |  |  |
| ALOX12 | NA | NA |  | | PTPRZ1 | | | 0.5137373335 | | | | 0.8741649102 |  |  |
| VCL | 0.9254636164 | 1.0506672102 |  | | CFL1 | | | 0.3702887624 | | | | 1.4859797658 |  |  |
| LBP | 0.2092530678 | 1.1131345531 |  | | LAMA2 | | | NA | | | | NA |  |  |
| SDC1 | 0.4570850423 | 1.8563410549 |  | | EEF1B2 | | | 0.5217741131 | | | | 0.7892094017 |  |  |
| VCAM1 | NA | NA |  | | TBXAS1 | | | NA | | | | NA |  |  |
| B3KXY9 | NA | NA |  | | MYL9 | | | 0.8584690788 | | | | 0.8911386622 |  |  |
| ORM2 | 0.1448458448 | 0.7126871992 |  | | AZGP1 | | | 0.0138130294 | | | | 0.6109645072 |  |  |
| ITIH2 | 0.9252587778 | 1.0168543538 |  | | ATP5A1 | | | NA | | | | NA |  |  |
| ITIH1 | 0.5934441041 | 1.0917610711 |  | | HELNASNA275 | | | NA | | | | NA |  |  |
| C4BPB | 0.2381303505 | 0.6368009181 |  | | PSMA3 | | | NA | | | | NA |  |  |
| PSMA4 | ▲ | ▲ |  | | CORO1A | | | NA | | | | NA |  |  |
| MSN | 0.8269943138 | 0.8404937088 |  | | GDI1 | | | NA | | | | NA |  |  |
| S100A4 | NA | NA |  | | YWHAB | | | 0.3034877370 | | | | 1.4577263639 |  |  |
| EEF1G | NA | NA |  | | SFN | | | 0.3848811128 | | | | 0.6381333970 |  |  |
| STOM | 0.8127959406 | 0.9194186425 |  | | THBS4 | | | 0.5351019727 | | | | 1.1962732319 |  |  |
| PON1 | 0.9843616703 | 0.9936793273 |  | | KRT9 | | | 0.5991717965 | | | | 0.7556160089 |  |  |
| KRT2 | 0.9557641696 | 1.0160135474 |  | | PIP4K2A | | | NA | | | | NA |  |  |
| FLT4 | NA | NA |  | | CD151 | | | NA | | | | NA |  |  |
| ATP5C1 | NA | NA |  | | IDH2 | | | NA | | | | NA |  |  |
| SERPINF1 | 0.1671525370 | 0.6075591694 |  | | MASP1 | | | 0.2573573546 | | | | 0.7866982430 |  |  |
| TAGLN2 | 0.7415696015 | 1.2136862248 |  | | THBS3 | | | NA | | | | NA |  |  |
| TALDO1 | NA | NA |  | | COMP | | | 0.4865747657 | | | | 1.1968188903 |  |  |
| SNCA | NA | NA |  | | SEPP1 | | | 0.1788557575 | | | | 0.7160103917 |  |  |
| HSPA9 | NA | NA |  | | CAMP | | | 0.4479269017 | | | | 0.8870783477 |  |  |
| COL18A1 | 0.8541054972 | 1.0974421633 |  | | GNAQ | | | NA | | | | NA |  |  |
| GP5 | NA | NA |  | | EMD | | | NA | | | | NA |  |  |
| MDH2 | NA | NA |  | | VASP | | | NA | | | | NA |  |  |
| PMEL | 0.9929657897 | 1.0025694373 |  | | CCT4 | | | NA | | | | NA |  |  |
| PTGDS | NA | NA |  | | RAB7A | | | NA | | | | NA |  |  |
| CSK | NA | NA |  | | BCAP31 | | | 0.4857799774 | | | | 0.6171586239 |  |  |
| BTD | 0.6862288941 | 1.1549717062 |  | | LUM | | | 0.7535616369 | | | | 1.0957186869 |  |  |
| AFM | 0.6142699105 | 0.8577128182 |  | | NDUFA8 | | | NA | | | | NA |  |  |
| VDAC2 | ▲ | ▲ |  | | PGD | | | NA | | | | NA |  |  |
| LIMS1 | 0.5283809619 | 0.5638804319 |  | | ARHGDIB | | | NA | | | | NA |  |  |
| Q59FJ0 | NA | NA |  | | Q53GF9 | | | NA | | | | NA |  |  |
| CAPZA1 | NA | NA |  | | YWHAG | | | 0.6329788445 | | | | 1.1539439599 |  |  |
| NAPA | ▲ | ▲ |  | | CALM2 | | | 0.1826349205 | | | | 0.6383866206 |  |  |
| MFAP2 | NA | NA |  | | YWHAE | | | 0.3982143581 | | | | 0.7659693714 |  |  |
| APOC4 | 0.6184526299 | 1.1026521302 |  | | TMSB4X | | | 0.4925830264 | | | | 0.5528146673 |  |  |
| PLTP | 0.4204425349 | 0.7007350291 |  | | RAB1A | | | NA | | | | NA |  |  |
| VCP | 0.6914834641 | 0.8956210085 |  | | GNB1 | | | NA | | | | NA |  |  |
| HADHB | NA | NA |  | | PPIA | | | 0.3573714818 | | | | 1.7910713061 |  |  |
| NAP1L1 | 0.3342445012 | 0.4923675347 |  | | FKBP1A | | | NA | | | | NA |  |  |
| ARPC4NATTLL3 | NA | NA |  | | B4E0X1 | | | 0.2603031561 | | | | 0.4821544566 |  |  |
| CD81 | * | * |  | | YWHAZ | | | 0.7636443860 | | | | 1.1184123463 |  |  |
| TPI1 | 0.5296724493 | 0.3727918234 |  | | ACTG1 | | | 0.2588966847 | | | | 1.9792950872 |  |  |
| MYL6 | 0.5600614912 | 0.6436993015 |  | | TPM4 | | | 0.5443406484 | | | | 1.3802974448 |  |  |
| ACTB | 0.6753932666 | 1.2428532273 |  | | ACTC1 | | | 0.4211540262 | | | | 1.7705325850 |  |  |
| PSMA6 | NA | NA |  | | TUBA1B | | | 0.8427947783 | | | | 1.1136076790 |  |  |
| RAB10 | NA | NA |  | | TUBA4A | | | 0.8712454591 | | | | 0.9139501581 |  |  |
| UBE2NL | NA | NA |  | | TUBB4B | | | 0.9214808899 | | | | 0.9677755993 |  |  |
| RAB14 | NA | NA |  | | HBB | | | 0.1208059213 | | | | 1.7938491646 |  |  |
| ACTR3 | NA | NA |  | | HBG1 | | | NA | | | | NA |  |  |
| ACTR2 | NA | NA |  | | HBA1 | | | 0.4669794701 | | | | 1.4130118839 |  |  |
| ACTR1A | NA | NA |  | | CCT2 | | | NA | | | | NA |  |  |
| ARF3 | 0.6780578973 | 1.3082666944 |  | | GSTO1 | | | 0.6841024860 | | | | 0.7171274424 |  |  |
| RAP1B | 0.8636210200 | 1.0957214407 |  | | RELN | | | NA | | | | NA |  |  |
| HSPE1 | NA | NA |  | | GPLD1 | | | 0.7257971188 | | | | 0.8723050318 |  |  |
| B4DMN1 | 0.5272801362 | 1.2698480342 |  | | B7Z9B8 | | | NA | | | | NA |  |  |
| DCD | NA | NA |  | | PTPRJ | | | 0.2285807261 | | | | 0.5870068815 |  |  |
| HSPG2 | 0.1486836381 | 1.2870255748 |  | | SPP2 | | | 0.7849658177 | | | | 0.9161794479 |  |  |
| MPP1 | NA | NA |  | | PRDX4 | | | NA | | | | NA |  |  |
| SLC25A3 | NA | NA |  | | MMRN1 | | | 0.9186232498 | | | | 0.9428437870 |  |  |
| CLTC | NA | NA |  | | ILK | | | 0.6112948349 | | | | 0.6693661042 |  |  |
| RHAG | NA | NA |  | | MADCAM1 | | | NA | | | | NA |  |  |
| AKAP12 | 0.2856787310 | 0.4144286869 |  | | IQGAP2 | | | NA | | | | NA |  |  |
| CFHR1 | 0.6058069561 | 1.1229096132 |  | | STIM1 | | | NA | | | | NA |  |  |
| YWHAH | 0.0638120321 | 0.4607093454 |  | | ALCAM | | | NA | | | | NA |  |  |
| PLP2 | NA | NA |  | | APOF | | | 0.7111826075 | | | | 0.8057284300 |  |  |
| CALD1 | 0.4374273906 | 8.0817620099 |  | | DCTN1 | | | NA | | | | NA |  |  |
| ITIH3 | 0.4426735778 | 0.8969041005 |  | | SELPLG | | | 0.1026792615 | | | | 0.7028367515 |  |  |
| FMOD | NA | NA |  | | CTTN | | | NA | | | | NA |  |  |
| PRDX1 | NA | NA |  | | FLOT2 | | | ▲ | | | | ▲ |  |  |
| DPT | 0.6137306701 | 0.8128267245 |  | | GAS6 | | | NA | | | | NA |  |  |
| LRP1 | 0.2446225045 | 0.4907447683 |  | | SPARCL1 | | | NA | | | | NA |  |  |
| LGALS3BP | 0.5721359636 | 0.8196446823 |  | | HABP2 | | | 0.5919554221 | | | | 1.0795869264 |  |  |
| Q59EU3 | NA | NA |  | | Q53H88 | | | NA | | | | NA |  |  |
| APOBR | NA | NA |  | | ITIH4 | | | 0.0652751933 | | | | 0.7404955935 |  |  |
| CGNL1 | NA | NA |  | | LTBP1 | | | 0.6823561659 | | | | 0.7953580813 |  |  |
| NEXN | NA | NA |  | | LTBP2 | | | 0.4981915710 | | | | 1.1989886694 |  |  |
| KCNMA1 | 0.4093245926 | 0.6932215411 |  | | LASP | | | NA | | | | NA |  |  |
| EFEMP1 | NA | NA |  | | KPNB1 | | | NA | | | | NA |  |  |
| B4DJ30 | 0.8285020225 | 0.9279420515 |  | | STXBP5 | | | NA | | | | NA |  |  |
| PDIA6 | 0.5252887064 | 1.7249383213 |  | | FAM21A | | | 0.4140590758 | | | | 0.8188095627 |  |  |
| PTGES3 | NA | NA |  | | VASN | | | * | | | | * |  |  |
| RCN1 | NA | NA |  | | C16orf46 | | | 0.0069162732 | | | | 0.6510058569 |  |  |
| TMED2 | NA | NA |  | | CASC4 | | | NA | | | | NA |  |  |
| RSU1 | 0.5391815221 | 0.7225786362 |  | | APOA5 | | | 0.9514114383 | | | | 0.9717490220 |  |  |
| MAPRE2 | 0.9956200125 | 0.9962744446 |  | | CSPG4 | | | 0.3558120923 | | | | 0.6815561580 |  |  |
| TGFBI | NA | NA |  | | FAM198B | | | NA | | | | NA |  |  |
| MAPRE1 | NA | NA |  | | PLXDC2 | | | 0.9154960657 | | | | 0.9125606562 |  |  |
| MYLK | NA | NA |  | | PI16 | | | 0.4725306154 | | | | 0.7389662537 |  |  |
| CD226 | NA | NA |  | | SEZ6L2 | | | NA | | | | NA |  |  |
| STXBP2 | NA | NA |  | | BNC2 | | | NA | | | | NA |  |  |
| ZYX | 0.1228284907 | 2.0637809271 |  | | CHST3 | | | NA | | | | NA |  |  |
| PRR4 | NA | NA |  | | ABI3BP | | | NA | | | | NA |  |  |
| ECM1 | 0.0309644947 | 0.6175360892 |  | | NRGN | | | NA | | | | NA |  |  |
| DBN1 | NA | NA |  | | NEO1 | | | NA | | | | NA |  |  |
| MAN2A1 | NA | NA |  | | PRG4 | | | 0.1630783195 | | | | 0.8223779207 |  |  |
| INF2 | NA | NA |  | | ATP2A3 | | | NA | | | | NA |  |  |
| LGALSL | NA | NA |  | | Q59EB6 | | | NA | | | | NA |  |  |
| HYDIN | 0.3337149436 | 0.8743994021 |  | | B4DID6 | | | 0.1899102570 | | | | 0.7206723380 |  |  |
| SVEP1 | 0.4025067899 | 1.3361225018 |  | | B7Z5W1 | | | NA | | | | NA |  |  |
| MIA3 | NA | NA |  | | A0A068LKQ2 | | | NA | | | | NA |  |  |
| A0A0G2JK05 | NA | NA |  | | B4DMS4 | | | 0.8754484646 | | | | 0.9001628728 |  |  |
| Q71SF7 | NA | NA |  | | B4DS05 | | | NA | | | | NA |  |  |
| FERMT3 | 0.8872584978 | 1.0822927329 |  | | OMD | | | 0.2374270253 | | | | 0.7120891981 |  |  |
| CAND1 | NA | NA |  | | TUBA1C | | | NA | | | | NA |  |  |
| TREML1 | 0.7285418495 | 0.9105776612 |  | | MENT | | | 0.3622711628 | | | | 0.6918343756 |  |  |
| HRNR | 0.8224682401 | 1.1621585264 |  | | TMEM109 | | | NA | | | | NA |  |  |
| PLXDC1 | 0.0019353803 | 0.3892718838 |  | | TMED9 | | | NA | | | | NA |  |  |
| CCDC50 | NA | NA |  | | COLEC11 | | | 0.3393111549 | | | | 0.8285581058 |  |  |
| GOLM1 | 0.3803585715 | 0.7127760706 |  | | ASPN | | | 0.4508787817 | | | | 0.6618919683 |  |  |
| PCSK9 | 0.2087526270 | 0.6909067627 |  | | CRTAC1 | | | NA | | | | NA |  |  |
| ANGPTL6 | NA | NA |  | | RTN4 | | | NA | | | | NA |  |  |
| LRRN4 | NA | NA |  | | SAR1A | | | NA | | | | NA |  |  |
| FCAMR | NA | NA |  | | RAB6B | | | NA | | | | NA |  |  |
| OIT3 | 0.3928323981 | 0.9594464084 |  | | SACM1L | | | NA | | | | NA |  |  |
| ST8SIA4 | 0.7024100581 | 1.0782140430 |  | | DKFZp686L04275 | | | NA | | | | NA |  |  |
| CFHR4 | * | * |  | | EHD3 | | | NA | | | | NA |  |  |
| CMTM5 | NA | NA |  | | ABRACL | | | NA | | | | NA |  |  |
| LTV1 | NA | NA |  | | Q59EA2 | | | NA | | | | NA |  |  |
| DKFZp686J1525 | 0.2390001463 | 0.3687198667 |  | | B7Z6C9 | | | 0.7347024087 | | | | 0.8012147832 |  |  |
| PGLYRP2 | 0.3479068254 | 0.8150092081 |  | | Q0ZCH6 | | | NA | | | | NA |  |  |
| ERBB2IP | NA | NA |  | | A0A0J9YX35 | | | NA | | | | NA |  |  |
| CNN2 | NA | NA |  | | A0A0J9YXX1 | | | NA | | | | NA |  |  |
| PARK7 | NA | NA |  | | IGLV1NA47 | | | NA | | | | NA |  |  |
| MGLL | NA | NA |  | | IGLV3NA10 | | | 0.6415887402 | | | | 0.9256256207 |  |  |
| APOL1 | 0.9389150054 | 0.9769619955 |  | | IGKV1NA17 | | | NA | | | | NA |  |  |
| NOGOC | 0.9829451428 | 1.0074709247 |  | | FBLN1 | | | 0.1223080265 | | | | 0.6071927260 |  |  |
| CADM1 | NA | NA |  | | BIN2 | | | 0.0923949106 | | | | 0.5215644709 |  |  |
| SEZ6L | 0.4261869370 | 0.7960387113 |  | | FETUB | | | NA | | | | NA |  |  |
| ROGDI | 0.3900262845 | 1.5123725387 |  | | TBK1 | | | NA | | | | NA |  |  |
| RAB1B | 0.9576402367 | 1.0314547992 |  | | CD84 | | | NA | | | | NA |  |  |
| SH3BGRL3 | 0.9675708018 | 0.9816804260 |  | | ATPIF1 | | | NA | | | | NA |  |  |
| TUBB1 | 0.7728946420 | 0.8457678087 |  | | SERPINA10 | | | 0.2378481274 | | | | 1.3678125583 |  |  |
| METTL7A | NA | NA |  | | EMCN | | | NA | | | | NA |  |  |
| PARVB | 0.6100662446 | 1.2068489028 |  | | HEG1 | | | 0.2097130766 | | | | 0.7415752000 |  |  |
| MUC5AC | NA | NA |  | | PACSIN2 | | | NA | | | | NA |  |  |
| GP6 | NA | NA |  | | CLEC11A | | | 0.4291933219 | | | | 0.7065147957 |  |  |
| CD248 | 0.6964804410 | 0.9037061605 |  | | HPSE | | | NA | | | | NA |  |  |
| APMAP | NA | NA |  | | VDAC3 | | | NA | | | | NA |  |  |
| EMILIN1 | NA | NA |  | | DHRS7 | | | NA | | | | NA |  |  |
| COLEC10 | 0.6755758869 | 1.0996773009 |  | | TMED7 | | | NA | | | | NA |  |  |
| FN1 | 0.6041186393 | 0.9308526247 |  | | TLN1 | | | 0.7232169848 | | | | 1.2321240143 |  |  |
| RTN3 | NA | NA |  | | HYOU1 | | | NA | | | | NA |  |  |
| TPM1 | ▲ | ▲ |  | | LYVE1 | | | 0.3839428074 | | | | 0.8812390775 |  |  |
| SERPINA1 | NA | NA |  | | HLANAC | | | 0.9226872626 | | | | 1.0581829667 |  |  |
| TNC variant protein | 0.7663723639 | 1.0519930941 |  | | APOE | | | NA | | | | NA |  |  |
| THBS1 | 0.9921807764 | 0.9960211782 |  | | TPM3 | | | 0.2976354310 | | | | 1.6285019120 |  |  |
| SPP1 | 0.8689339774 | 1.0529791839 |  | | HLANAB | | | NA | | | | NA |  |  |
| V<kappa>1 | 0.5334014285 | 1.4813333455 |  | | A2NUT2 | | | 0.8037865174 | | | | 1.1878662148 |  |  |
| V<kappa>3 | 0.4301878472 | 1.3501567632 |  | | A2NW97 | | | NA | | | | NA |  |  |
| IGLV4NA69 | 0.2691183875 | 0.8461665388 |  | | HELNA214 | | | 0.5961747160 | | | | 0.8909983799 |  |  |
| IGLV10NA54 | NA | NA |  | | IGHV3NA72 | | | 0.4279439734 | | | | 0.6741779806 |  |  |
| IGLV2NA14 | NA | NA |  | | ALB | | | NA | | | | NA |  |  |
| IGLV3NA9 | NA | NA |  | | IGKV1NA12 | | | NA | | | | NA |  |  |
| IGLV3NA1 | NA | NA |  | | IGHG3 | | | 0.9485810729 | | | | 1.0324383699 |  |  |
| IGHA2 | 0.3363014584 | 0.3468908094 |  | | IGHG1 | | | 0.3716311333 | | | | 1.1550591037 |  |  |
| IGKV2DNA40 | NA | NA |  | | IGHM | | | 0.7436134847 | | | | 0.8386370789 |  |  |
| IGKV2DNA24 | NA | NA |  | | IGKV1DNA33 | | | 0.3440862798 | | | | 0.3142461172 |  |  |
| IGKV A18 | 0.4517245917 | 1.2408696894 |  | | IGHM | | | 0.3131921980 | | | | 1.2658422817 |  |  |
| IGKV3DNA15 | NA | NA |  | | SAA2NASAA4 | | | 0.4480661142 | | | | 1.3249776871 |  |  |
| NCAM1 | 0.3177434544 | 0.2637713748 |  | | IGKV1NA16 | | | NA | | | | NA |  |  |
| A0A0X9T7T4 | NA | NA |  | | IGHV3NA74 | | | 0.5433873472 | | | | 1.3485945989 |  |  |
| A0A0X9UWK7 | 0.1439993788 | 0.6490028267 |  | | IGKV3DNA20 | | | 0.1678440852 | | | | 1.5217946219 |  |  |
| A0A0X9UWL5 | 0.1868300545 | 1.4197251864 |  | | A2J1M4 | | | NA | | | | NA |  |  |
| A0A109PP82 | NA | NA |  | | A2J1M8 | | | NA | | | | NA |  |  |
| A0A109PSY4 | NA | NA |  | | A2J1N6 | | | NA | | | | NA |  |  |
| A0A125QYY9 | 0.7786277267 | 0.8320543093 |  | | A2KBC6 | | | 0.6209796343 | | | | 0.8821489636 |  |  |
| A0A5E4 | 0.2849990139 | 1.5778987091 |  | | KNG1 | | | 0.4253498780 | | | | 1.1777546183 |  |  |
| A8K008 | 0.4562266154 | 0.6932904119 |  | | C1R | | | 0.5414456464 | | | | 0.9302131319 |  |  |
| A8K0D8 | 0.0227148395 | 0.5240117346 |  | | gcysNA20 | | | NA | | | | NA |  |  |
| A8K5T0 | NA | NA |  | | C4B | | | NA | | | | NA |  |  |
| S6AWE6 | 0.3313977995 | 0.8678194821 |  | | S6BGD6 | | | 0.3141011535 | | | | 1.4784130951 |  |  |
| S6AWF4 | 0.0697129663 | 0.4020573354 |  | | S6BGE0 | | | 0.2847364755 | | | | 1.3068009727 |  |  |
| S6B291 | NA | NA |  | | IGK | | | 0.1520547850 | | | | 1.5433742522 |  |  |
| DKFZp686I15196 | NA | NA |  | | DKFZp686N02209 | | | NA | | | | NA |  |  |
| B1N7B8 | NA | NA |  | | B4DR99 | | | NA | | | | NA |  |  |
| B1N7B9 | 0.6022303450 | 1.2577423186 |  | | B4DTY9 | | | 0.1801890399 | | | | 1.6106634024 |  |  |
| B2R582 | 0.3229659163 | 0.7531283285 |  | | B4DUI7 | | | 0.6022953317 | | | | 1.3727452027 |  |  |
| B4DWA6 | NA | NA |  | | B4DVJ0 | | | NA | | | | NA |  |  |
| B2R8I2 | 0.1123581192 | 0.6707495481 |  | | B4DXW3 | | | NA | | | | NA |  |  |
| B2RBZ5 | 0.3006870040 | 0.5819314121 |  | | B4E1B2 | | | 0.1331077937 | | | | 0.6974343404 |  |  |
| B2RDY9 | 0.9619433934 | 0.9756243160 |  | | B4E1D8 | | | 0.0925386244 | | | | 0.3445508256 |  |  |
| B4DF70 | 0.3043536105 | 1.3884312402 |  | | B4E1Z4 | | | 0.1380608308 | | | | 0.6786822144 |  |  |
| IGK@ | 0.9252575111 | 0.9588825832 |  | | B4E344 | | | NA | | | | NA |  |  |
| IGL@ | 0.3426831001 | 0.8368242200 |  | | B7Z1F8 | | | 0.7283045213 | | | | 0.7473554519 |  |  |
| Q6XMI6 | 0.5713291098 | 0.7732299880 |  | | B7Z539 | | | 0.7506326283 | | | | 0.8109943870 |  |  |
| TPM1 | 0.5153225533 | 0.6750312783 |  | | B7Z992 | | | NA | | | | NA |  |  |
| Q86TT1 | 0.3724398407 | 1.3607274242 |  | | Q9UL70 | | | NA | | | | NA |  |  |
| HLANAB | NA | NA |  | | Q9UL78 | | | 0.1865472041 | | | | 0.5195410325 |  |  |
| IGL@ | 0.3447798332 | 0.9422593789 |  | | P04431 | | | NA | | | | NA |  |  |
| SPARCL1 | 0.5411966145 | 0.8717562406 |  | | Q9UL82 | | | 0.6649296594 | | | | 0.9302304775 |  |  |
| IGL@ | 0.4077527324 | 0.4913985873 |  | | Q9UL83 | | | 0.8689867467 | | | | 0.9548071665 |  |  |
| Q96JD0 | NA | NA |  | | Q9UL88 | | | 0.6500242330 | | | | 1.2844492923 |  |  |
| Q96K68 | 0.4076619209 | 0.7914696554 |  | | C4B | | | 0.6352189370 | | | | 1.2232722593 |  |  |
| Q9NPP6 | 0.3260406613 | 0.8141846694 |  | | R4KXX0 | | | NA | | | | NA |  |  |
| Q9P1C5 | 0.1660624784 | 1.5703855906 |  | | AMBP | | | 0.2421734449 | | | | 2.2502977970 |  |  |
| Q9UE89 | NA | NA |  | | ZIK1 | | | NA | | | | NA |  |  |
| APOB | 0.7086653456 | 0.9552484439 |  | | Q06AK0 | | | 0.2604665784 | | | | 0.6946884267 |  |  |
| C1S | 0.3159393210 | 0.7006298066 |  | | Q8TCD0 | | | 0.3796400682 | | | | 0.8015694977 |  |  |
| FGG | 0.6246107763 | 1.2952343002 |  | | IGH@ | | | NA | | | | NA |  |  |
| SAA1 | 0.3812588077 | 4.1217047181 |  | | Q12984 | | | NA | | | | NA |  |  |
| FAM63A | NA | NA |  | | F5 | | | 0.3022149302 | | | | 2.1806216195 |  |  |
| GC | 0.3335494628 | 0.8019907075 |  | | Q2HZY9 | | | 0.9687377876 | | | | 0.9764799761 |  |  |
| IL7R | * | * |  | | HLANAB | | | * | | | | * |  |  |
| TPM1 | 0.5110583523 | 1.4755132743 |  | | IGL@ | | | 0.9354946023 | | | | 1.0122354323 |  |  |
| GP1BA | NA | NA |  | | DKFZp686A0568 | | | 0.8984883593 | | | | 1.0448136796 |  |  |
| CSF1 | NA | NA |  | | DKFZp686O1553 | | | 0.9279041734 | | | | 1.0285247338 |  |  |
| HLANAC | NA | NA |  | | V1NA13 | | | NA | | | | NA |  |  |
| MYL6 | NA | NA |  | | IGLV3NA25 | | | NA | | | | NA |  |  |
| HLANAA | NA | NA |  | | TPM2 | | | 0.9120188104 | | | | 1.0775035321 |  |  |
| NDRG4 | ▲ | ▲ |  | | scFv | | | NA | | | | NA |  |  |
| TPM1 | 0.5035501623 | 1.7498964591 |  | | IGL@ | | | 0.2000136396 | | | | 0.8343943236 |  |  |
| PDIA3 | NA | NA |  | | IGH@ | | | 0.3123003466 | | | | 1.4725330477 |  |  |
| ITIH1 | 0.0129672357 | 0.2721010825 |  | | IGL@ | | | NA | | | | NA |  |  |
| TPM3 | NA | NA |  | | DKFZp686K06110 | | | NA | | | | NA |  |  |
| ZNF207 | NA | NA |  | | DKFZp686M0562 | | | 0.7686112230 | | | | 1.1194355633 |  |  |
| TPM4 | NA | NA |  | | DKFZp686G11190 | | | 0.6195073390 | | | | 0.7141306690 |  |  |
| APOC4NAAPOC2 | 0.2632581576 | 0.5993388617 |  | | DKFZp686C15213 | | | NA | | | | NA |  |  |
| MMP13 | NA | NA |  | | DKFZp686O16217 | | | NA | | | | NA |  |  |
| VWF | 0.9382256289 | 0.9774899672 |  | | DKFZp686P15220 | | | NA | | | | NA |  |  |
| HLANAA | 0.5170285538 | 1.3223936789 |  | | DKFZp686K18196 | | | 0.1944316967 | | | | 0.4909074876 |  |  |
| HLANAB | NA | NA |  | | DKFZp686I04196 | | | 0.9463627531 | | | | 1.0100230070 |  |  |
| YWHAQ | NA | NA |  | | FBN1 | | | * | | | | * |  |  |
| CD82 | NA | NA |  | | MYH9 | | | 0.8744310459 | | | | 1.0867569577 |  |  |
| CALR | 0.0985223664 | 0.8184161475 |  | | IGFALS | | | 0.0005309625 | | | | 0.3184264259 |  |  |
| CANX | 0.8926518633 | 1.0237297028 |  | | PDIA3 | | | 0.3867806327 | | | | 0.6907735865 |  |  |
| PSMB8 | NA | NA |  | | FCER1G | | | NA | | | | NA |  |  |
| PSMA5 | NA | NA |  | | PPIF | | | NA | | | | NA |  |  |
| PSMB4 | NA | NA |  | | SERPINB1 | | | NA | | | | NA |  |  |
| PSMB6 | 0.3574570454 | 0.7915616293 |  | | EEF1D | | | NA | | | | NA |  |  |
| CD34 | NA | NA |  | | PRDX6 | | | NA | | | | NA |  |  |
| SERPINA4 | 0.1334178619 | 0.2976237745 |  | | PRDX3 | | | NA | | | | NA |  |  |
| S6BGD4 | 0.0494440185 | 1.2414402414 | |  | | | ATP5D | | NA | NA | | | |  |

Note: Asterisk “*” represents the detected protein in benign PNs group; Solid triangle “▲” represents the detected protein in malignant PNs group. NA: Not applicable.

**

Table S3. Top 20 proteins in benign PNs.**

**

Table S4. Top 20 proteins in malignant PNs.**
